# Positive psychology 4.0: a science of consequence for a world that can no longer wait

**DOI:** 10.3389/fpsyg.2026.1726065

**Published:** 2026-07-08

**Authors:** Llewellyn E. van Zyl

**Affiliations:** 1 Psynalytics – AI Powered People Analytics, Eindhoven, Noord Brabant, Netherlands; 2 Optentia Research Unit, North-West University, Vanderbijlpark, Gauteng, South Africa

**Keywords:** AI-IARA framework, human-AI collaboration, positive ethics, positive psychology 4.0, precision psychology, regenerative wellbeing

## Abstract

Positive psychology’s existing frameworks are not keeping pace with the convergent crises and technological transformations reshaping human experience. This perspective paper proposes the Fourth Wave of Positive Psychology (PP 4.0): a meta-integrative reconceptualization of the field through four simultaneous, interdependent shifts. First, the regenerative revolution repositions wellbeing’s basic unit of analysis from individual optimization to the health of life-sustaining systems, recognizing that personal flourishing cannot be separated from systemic regeneration. Second, the precision revolution replaces nomothetic averaging with idiographic methodologies, including digital phenotyping and person-specific models, to capture how wellbeing emerges uniquely within each person’s context. Third, the computational revolution integrates human-AI collaboration into research and practice through human-in-the-loop protocols that augment human wisdom without replacing it. Fourth, the positive ethics revolution establishes governance frameworks that prevent algorithmic bias, preserve human agency under algorithmic conditions, and ensure equitable access to emerging technologies. The paper further identifies three emergent challenges arising from the convergence of these revolutions: the algorithmic capture of wellbeing definitions, the agency-optimization paradox addressed by the AI-IARA framework, and the collision of temporal scales from real-time intervention to intergenerational stewardship. Drawing on parallel innovations in precision medicine, digital twin technology, biosensor monitoring, and network neuroscience, the paper articulates a methodological architecture for conducting PP 4.0 research, demonstrates its integration through the Adaptive Twin-Led Assessment System (ATLAS), and projects concrete developments through 2035. This framework is presented as both a scientific contribution offering testable propositions and a normative call to reorient the field’s purpose. PP 4.0 does not extend positive psychology. It reimagines the discipline as one capable of addressing existential threats, honoring epistemological pluralism, and advancing collective regenerative flourishing rather than extracting individual happiness from depleting systems.

## Introduction: the inadequacy of incremental thinking

Positive psychology now has accumulated more knowledge about what makes individuals flourish than any other discipline at any other point in human history. It has established new pathways to meaning (Martela and Steger, 2023), mapped the architecture of hope (Colla et al., 2022), and developed interventions for resilience with increasing precision (Liu et al., 2022). And yet, the systems that make flourishing possible (i.e., stable ecologies, functioning democracies, equitable economies, and cohesive communities) are paradoxically deteriorating faster than the field can publish. This paradox is therefore not a result of insufficient knowledge or insight, but is rather a paradox of insufficient framing. A science that aims to perfect the study and development of individual wellbeing, while the conditions for collective wellbeing collapse around them, has not failed in its methods. It has failed in its imagination.

As such, positive psychology stands at yet another inflection point. One that does not demand an incremental evolution of its scope or methods but rather a fundamental reimagining of its purpose (Van Zyl et al., 2024). In 2026, humanity is confronted with existential threats and challenges which range from escalating wars, cascading ecological crises, and accelerating inequality to massive technological disruptions to our way of being and the quiet erosion of democratic values. These existential threats render positive psychology’s approach to making incremental advancements to the science of wellbeing not only insufficient but morally indefensible (Steger, 2025). Yet recent attempts to chart the field’s future trajectory have failed to match the scale of transformation required to address these pressing challenges (Lomas et al., 2021; Ryff, 2024; Van Zyl et al., 2024; Wissing, 2022). Throughout its history, the development of the theoretical and empirical foundations of positive psychology has always emerged out of extreme criticisms and dynamic uncertainty. The September 11 attacks (2001), the global financial crisis (2008), and the COVID-19 pandemic (2020) have each radically reconfigured the conditions of what constitutes “the good life.” Further, challenges within the discipline like the controversies around the critical positivity ratio (Friedman and Brown, 2018), the debunking of the happiness pie (Brown and Rohrer, 2020), the “WEIRD” cultural bias underpinning the majority of its empirical foundations and the jingle-jangle fallacies centered around it (Van Zyl et al., 2024) have all forced the field to constantly change and adapt.

But what distinguishes this present moment is not the intensity of crisis or the level of criticisms levelled against it but rather its structure. Contemporary challenges are characterized by unprecedented speed of feedback loops, by the way they cut across individual, social, technological, and ecological levels simultaneously, and by their capacity to interact and amplify one another in ways that exceed what previous frameworks were designed to address (Steger, 2025). It is this structural convergence of challenges that renders its approach for incremental changes and adaptation to be insufficient. Part of the problem is also institutional rather than purely epistemic: the gap between research and its real-world implementation, the insufficient translation of positive psychology knowledge into policy and practice, and the persistent misrepresentation of the field in popular discourse all constrain the discipline’s impact in ways that cannot be resolved through theoretical reconceptualization alone (Wissing, 2022). However, these institutional limitations are themselves compounded by theoretical architecture that has not kept pace with the structural complexity of contemporary challenges. Both levels of diagnosis must be addressed for the field to grow. But the present paper is primarily concerned with the theoretical level while acknowledging the indispensability of the institutional one.

It is against this backdrop that various individuals and research teams have tried to reorientate the field in a new direction (cf. Wissing, 2022 for an overview). But even here, attempts have fallen short. Perhaps the most notable attempt in recent years to chart the field’s future direction that has fallen short was that of Lomas et al. (2021). In their attempt to reorientated the field, Lomas et al.’s (2021, p. 1) proposed a “third wave” framework for broadening positive psychology’s scope “toward [more] complexity” emphasized systems thinking, contextual approaches to wellbeing, cultural and linguistic sensitivity, ethical practices, and expanded methodological pluralism. However, from its outset this framework suffered from three limitations that render it inadequate as a guide for the field’s future.

### The three challenges of the third wave conceptualization

First, the third wave conception is fundamentally *descriptive rather than being transformative* (Wissing, 2022). Whilst it was valuable for cataloguing current trends (at the time), these proposed ‘new’ directions largely repackaged existing concepts from adjacent psychology domains (e.g., systems thinking from community psychology and complexity science; contextual approaches from ecological psychology; cultural sensitivity from cross-cultural psychology) (Wissing, 2022). Further, these concepts were listed as aspirational directions without specifying how to integrate them into a unified theoretical framework or how to operationalize them through concrete methodological innovations (Van Zyl et al., 2024). The result is a framework that identifies “complexity” without providing the analytical tools required to navigate it. One that documents the importance of cultural diversity without resolving epistemological tensions between Western and indigenous ways of knowing (Van Zyl, 2025). And one that acknowledges systemic influences without fundamentally repositioning the individual within broader ecological and social systems (Steger, 2025; Wissing, 2022). In essence, the third wave describes what positive psychology *should* attend to but offers no transformative roadmap for how the field must fundamentally restructure its theories, methods, or practices to do so.

Second, and perhaps more critically, the third wave framework was already largely *overtaken by developments already underway at the time of its publication*. By 2021, many of its defining features had already been absorbed into mainstream positive psychology research (cf. Wang et al., 2023; Wissing, 2022). A recent bibliometric review of positive psychological research shows a marked rise in qualitative and mixed-method studies, network and systems-informed approaches (such as collective flow and ecological wellbeing), and a surge in work developing more indigenous and non-Western measures of flourishing (Wang et al., 2023). The field had already begun to move beyond the individualistic focus Lomas and colleagues sought to correct and had already shown signs of incorporating more systems perspectives in understanding positive psychological phenomena (Wang et al., 2023). For example, research on flow has expanded its focus away from individual states to more group and collective dynamics (Peifer et al., 2022). Meta-analytic studies highlighted a significant amount of studies focused on linking connection to nature with human wellbeing through different ecological relationships that are consistent with a mesosystem to macrosystem orientation (Zeng et al., 2025). These literatures show that positive outcomes are coupled to environmental and group processes, not only to individual traits, which again aligns with the third-wave thesis. The need for more cultural and linguistic approaches in positive psychology has also long been part of its discourse (Van Zyl et al., 2024). In 2003, Flores and Obasi (2003) already coined the term “cross-cultural positive psychology” and recent bibliometric reviews show that there has been a rapid rise in non-Western approaches to wellbeing (e.g., Fadiji et al., 2024; Mad Plume et al., 2024). These developments move beyond just adapting Western models for local applicability but are fundamentally geared at developing indigenous positive psychologies (Christopher and Howe, 2014; Lopez, 2011; Lambert et al., 2015). Further, digital and computational strands of research started with Sander (2011) and has now bloomed into its own dedicated field called “positive computing” and more recent “computational positive psychology” (Hou et al., 2025).

This is not to diminish the third wave’s contribution. Synthesizing dispersed tendencies within a discipline and giving them conceptual coherence is valuable scholarly work, and Lomas et al. (2021) accomplished this effectively for the moment in which they wrote (Van Zyl et al., 2024). The problem is not that the synthesis was wrong but that it was already behind. Fundamental questions about the nature of flourishing, the structure of character strengths, and the dynamics of flow remain methodologically complex and theoretically open, and these questions will not be resolved by frameworks calibrated to the conditions the third wave assumed. What the field needed was not a catalogue of where it had already arrived but an architecture for where it must go next. That architecture became urgent because, since Lomas et al.'s (2021) publication, the field has been confronted with transformative developments that altered the science of wellbeing in ways they could not have anticipated.

For example, Steger (2025) conceptualized a regenerative positive psychology by reframing the basic unit of analysis from individual wellbeing to the health of life-sustaining systems. Van Zyl and Dik (2025) demonstrated how precision approaches using person-specific methodologies and machine learning can escape the “science of averages” problem that has plagued psychology. Thakkar et al. (2024) revealed artificial intelligence’s capacity to augment wellbeing interventions through continuous monitoring and adaptive personalization. Indigenous scholars are articulating genuinely pluralistic epistemologies that challenge Western psychological frameworks at their ontological foundations (Wissing, 2022). These developments represent not incremental advances within the third wave but qualitative ruptures demanding entirely new conceptual frameworks.

Third, and most fundamentally, the third wave framework also *lacks moral urgency* (Wissing, 2022). A stance that merely “broadens” positive psychology without fundamentally reorienting its purpose fails to meet the emerging, current and historical challenges it faces. Climate catastrophes, rising inequality, collapses in democracy, wars and technological disruptions etc. create existential threats that demand radical changes to the discipline and not a slight expansion of its scope. As Ryff (2024) demonstrated, wellbeing is becoming a privilege of the wealthy which is fundamentally undermining claims that positive psychology offers universal pathways to flourishing. Any adequate framework must confront rather than accommodate these realities.

In essence, the third wave framework largely reframes the field’s scope without actually redefining its moral intent. It invites contextual sensitivity and methodological pluralism but leaves purpose and governance behind. Without clearly articulating its telos, like the protection of life-sustaining systems, intergenerational justice, and equitable access to wellbeing (Steger, 2025), the field risks becoming even more descriptive rather than directive in nature (Wissing, 2022). In other words, it will be able to map and describe complexity but not mandate change. A morally urgent science must set thresholds for harm, design obligations for institutions, triage priorities for vulnerable populations, and ethical safeguards for technologies that now mediate human experience (Van Zyl, 2026d). It must expand the meaning of validity to include ecological sustainability and social justice and ensuring that an intervention or measure is considered credible only when it is both methodologically sound and ethically defensible. So, despite the call for stronger ethics (Lomas et al., 2021), the third wave, provides no architecture for the field’s moral intent.

But introducing moral urgency as a criterion for evaluating the theoretical framework of positive psychology may raise legitimate concerns about blurring the boundary between scientific analysis and normative advocacy (Van Zyl, 2025). The argument here is not that moral urgency, in and of itself, should replace the epistemic and methodological criteria for evaluating research quality. But rather, for a discipline whose explicit aim is to understand and cultivate human flourishing, normative reflection on the purposes and consequences of research constitutes a complementary, and not a competing, evaluative dimension (Alexandrova, 2017; Slife and Richardson, 2008). The question of telos, that is, whether positive psychology should articulate explicit goals regarding the protection of life-sustaining systems or intergenerational justice (Steger, 2025), can be treated as a separate level of reflection that complements rather than replaces epistemic argumentation. Whether such a telos is a strength or a potential threat to theoretical and methodological pluralism is a question the field is invited to debate this openly.

But before articulating the alternative, a plausible counter position also deserves to be acknowledged. And that is that the priority for positive psychology, as a subdiscipline still working toward epistemic consolidation, should be to strengthen its existing own theoretical and methodological coherence rather than just to keep pursuing yet another reconceptualization (Van Zyl, 2025). Organizing and deepening the foundations of the field by defining a stable epistemic architecture that integrates the insights of all previous waves represents a legitimate alternative developmental path which we need to explore. This alternative deserves serious consideration (Van Zyl et al., 2024), but it is ultimately not enough to address the challenges that has been outlined above (Van Zyl, 2025; Wissing, 2022). The structural convergence of the ecological, technological, and social crises society faces creates conditions that differ not merely in degree but in kind from those under which previous waves were formulated. A strategy of gradual epistemic consolidation, however valuable it may be during times of stability (Van Zyl and Rothmann, 2022), risks leaving the field permanently lagging behind transformations that are reshaping the very phenomena it studies. But whichever road the discipline takes to respond to these challenges, will ultimately depend on the research program it generates rather than on the ambition of how this approach is framed.

### Three emerging conditions reshaping positive psychology’s relationship to its foundations

But beyond these three limitations, the landscape on which any adequate framework must now be built has itself shifted in ways that compound the inadequacy of the existing approaches (Van Zyl, 2025). Three emergent conditions, none of which existed at the scale or in the form they now take when the third wave was formulated, are reshaping the fundamental relationship between positive psychology and the phenomena it studies.

The first condition is the *quiet transfer of epistemic authority over the definition of flourishing from human communities to algorithmic systems* (Van Zyl, 2026a). Fitness applications now operationalize wellbeing as steps and heart rate variability. Mental health platforms reduce it to symptom scores and session engagement (Van Zyl, 2026b). Wellbeing chatbots used by hundreds of millions of people define therapeutic success through user retention metrics (Van Zyl, 2026a). These are not definitions of wellbeing. They are measurable proxies selected because they are easy to quantify (Van Zyl, 2026b). But once such proxies become embedded in systems mediating the daily experience of billions, they cease to function as proxies and become the operational definition of flourishing, not because they are correct but because they are ubiquitous (Van Zyl, 2026a). Shin (2026) describes this process as “algorithmic truth”: the mechanism through which AI systems construct and reconfigure the epistemic conditions under which claims about human experience are validated. Previous waves of positive psychology debated whose definition of flourishing should prevail. The field must now confront whether the definition remains in human hands at all (Coeckelbergh, 2025).

The second condition is the *accumulating evidence that AI-mediated psychological support may systematically erode the very capacities it claims to enhance* (Van Zyl, 2026b). This is not a speculative concern. Gerlich (2025) found that cognitive offloading mediates a negative relationship between AI tool usage and critical thinking, particularly among younger populations. Neuroimaging research demonstrates that brain connectivity systematically reduces in synchronization with the degree of external AI support, with AI-assisted participants showing the weakest neural coupling in frequency bands associated with internal attention and working memory (Kosmyna et al., 2025). Kim et al. (2026) identified a predictable trajectory from rational efficiency-seeking through progressive dependency toward “cognitive surrender,” the uncritical adoption of algorithmic outputs even following recognized errors. Jose et al. (2025) showed that AI-mediated coping can paradoxically exacerbate the distress it was designed to relieve, generating feedback loops of increasing reliance. These findings converge on a single pattern: the infrastructure being built to optimize human flourishing may be incompatible with the experience of flourishing itself. A science of wellbeing that does not account for this paradox is building on foundations it is simultaneously eroding. The AI-IARA framework (Van Zyl, 2026a) provides the most developed response to this challenge, identifying six irreducible psychological capacities, Awareness, Interpretation, Intention, Action, Relational Agency, and Autonomy, that either strengthen or atrophy depending on how AI systems are designed and used. These capacities cascade: when Awareness erodes, Interpretation is outsourced, Intention drifts, Action defaults to algorithmic recommendation, Relational Agency is displaced by AI companionship, and Autonomy progressively surrenders (Van Zyl, 2026a). Any framework claiming to advance human flourishing through AI-augmented methods must demonstrate that it strengthens rather than depletes these capacities.

The third condition is the *collision of temporal scales that no existing psychological framework integrates*. Real-time adaptive interventions operate at the scale of minutes. Digital phenotyping and person-specific models unfold over weeks to months. Developmental trajectories span years but regenerative systemic change requires decades. Intergenerational justice, as now articulated within wellbeing science (Steger, 2025), extends moral consideration across centuries. These timescales are not merely different in magnitude. They can produce contradictory prescriptions. An intervention that optimizes momentary positive affect may undermine the distress tolerance required for long-term resilience (Van Zyl et al., 2024). A technology delivering measurable improvements in this generation’s flourishing metrics may deplete the psychological or ecological resources available to the next (Van Zyl, 2026a). A science of wellbeing that operates at only one temporal scale is, at best, incomplete. At worst, it optimizes the present at the expense of the future.

These three conditions share a common structure: in each case, the tools positive psychology is building to understand flourishing are simultaneously reshaping what flourishing means. This is not a problem that more research within existing paradigms can solve. It is a problem that requires rethinking what a paradigm is for.

### Toward positive psychology 4.0: a meta-integrative response

Taken together, these three limitations of the third wave framework, its descriptive rather than transformative character, its absorption by developments already underway, and its absence of moral urgency, combined with three emergent conditions reshaping the field’s operating environment, the algorithmic capture of wellbeing definitions, the systematic erosion of human agency through the very tools designed to enhance it, and the unresolved collision of temporal scales from milliseconds to generations, demand not a third wave but a fourth. This paper proposes Positive Psychology 4.0 (PP 4.0) as a reconceptualization of the field through four simultaneous, interdependent shifts that together constitute a meta-integrative transformation. The *regenerative revolution* repositions wellbeing within life-sustaining systems, moving from individual optimization to ecosystem regeneration (Steger, 2025). The *precision revolution* escapes nomothetic averaging through idiographic methodologies, digital phenotyping, and person-specific approaches to wellbeing (Van Zyl and Dik, 2025). The *computational revolution* positions human-AI collaboration as fundamental to research and practice while maintaining human agency through human-in-the-loop governance (Hou et al., 2025). The *positive ethics revolution* establishes frameworks to govern emerging technologies, preventing algorithmic amplification of existing inequalities and preserving the psychological capacities identified by the AI-IARA framework as essential for flourishing under algorithmic conditions (Van Zyl, 2026d; Van Zyl, 2026a).

Further, PP 4.0 draws on parallel revolutionary developments in medical science and neuroscience that provide both methodological templates and cautionary tales. Precision medicine has demonstrated how integrating genomics, proteomics, and computational modelling enables truly personalized interventions (Alsaedi et al., 2025). Digital twin technology is creating dynamic virtual replicas of patients that enable predictive modelling and treatment optimization (Vallée, 2024). Wearable biosensors provide continuous multimodal monitoring, revealing patterns invisible to traditional assessment (Duan et al., 2025). Network neuroscience demonstrates how brain function emerges from complex system interactions rather than isolated regional activations (Barabási et al., 2023). Quantum computing promises to revolutionize pattern recognition and optimization in ways classical computing cannot achieve (Fairburn et al., 2025). These parallel developments are not peripheral to positive psychology but constitute the methodological foundation upon which PP 4.0 must be constructed.

As such, this paper proceeds in three sections. First, the paper articulates the four revolutions underpinning PP 4.0 by demonstrating how each fundamentally transforms positive psychology’s theoretical and methodological foundations. Second, the paper presents a comprehensive methodological architecture for PP 4.0 by detailing the specific techniques, technologies, and approaches required to take the field forward. Finally, the paper projects forward 10 years by articulating concrete predictions about how positive psychology will operate in 2035^1^.

## The four convergent revolutions of positive psychology 4.0

**Figure 1:**
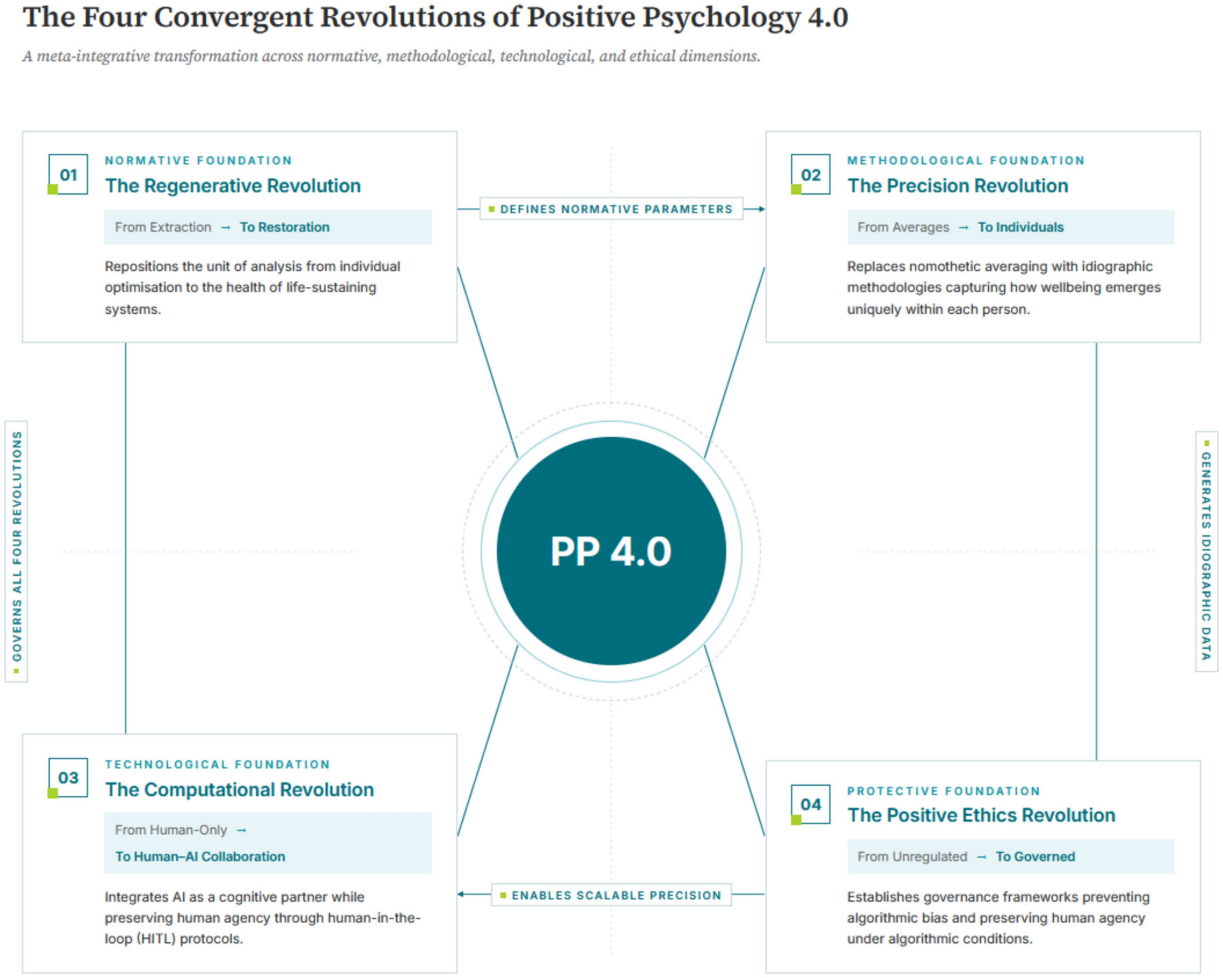
The four convergent revolutions of positive psychology 4.0.

To address this first objective, the paper begins by outlining the four convergent revolutions that together define PP 4.0 (cf. Figure 1 for a visual summary). These four revolutions are not sequential stages in the field’s evolution, but simultaneous paradigm shifts that, taken together, are fundamentally redefining positive psychology’s scope, methods, and moral purpose. They address the field’s most critical limitations: its extractive relationship with broader systems, its methodological reliance on nomothetic averaging, its human-only approach to knowledge generation, and its insufficient ethical frameworks for emerging technologies (Van Zyl, 2025). Each revolution is both independent and interdependent on each other. The regenerative revolution provides PP 4.0’s normative foundation by establishing that flourishing cannot be separated from systemic health. The precision revolution provides its methodological foundation by enabling an understanding of how systemic factors manifest in individual lives and how to develop it from a person-specific perspective. The computational revolution provides its technological foundation by augmenting human wisdom with AI capabilities. The positive ethics revolution provides its protective foundation by establishing the safeguards needed to prevent the technological amplification of existing inequalities. Together, these four revolutions do not constitute an extension or incremental expansion of positive psychology’s scope but rather lead to a complete reconceptualization of the study of human flourishing.

Significant epistemological tensions, however, exist within this architecture. The most salient is between the regenerative revolution’s systemic epistemology, which privileges macro-level analysis, and the precision revolution’s idiographic epistemology, which radicalizes micro-level inquiry. This tension has not been resolved fully. The present argument is that it is productive rather than paralyzing: systemic analysis identifies the conditions under which individuals must construct their wellbeing, while idiographic analysis reveals how they actually do so. Neither level is reducible to the other, and both are necessary for a comprehensive science of flourishing (Molenaar, 2004; Steger, 2025). A related tension exists between the precision revolution’s rejection of nomothetic averaging and the computational revolution’s reliance on algorithms trained on large datasets. This tension is addressed by distinguishing between nomothetic generalizations imposed as universal laws and nomothetic patterns extracted from aggregated idiographic profiles. The latter represents not a continuation of traditional nomotheticism but a new form of knowledge generation where general patterns emerge bottom-up from the systematic synthesis of person-specific findings (Van Zyl and Dik, 2025).

These functional interdependencies and epistemological tensions are not merely architectural features of the framework. They are the mechanism through which the three emergent conditions identified in the preceding section arise. The epistemic authority problem, the question of who retains the right to define flourishing under algorithmic conditions, sits at the intersection of all four revolutions: the regenerative revolution determines whose systems count, the precision revolution determines whose data shapes models, the computational revolution determines whose algorithms process that data, and the ethics revolution must govern the values encoded in each. The agency-optimization paradox, addressed by the AI-IARA framework (Van Zyl, 2026a), emerges specifically where precision monitoring meets computational augmentation, where the capacity to observe and intervene upon wellbeing at unprecedented resolution encounters the evidence that such observation and intervention may erode the capacities wellbeing requires. The temporal integration problem surfaces where regenerative thinking, with its generational timescale, meets the real-time logic of adaptive intervention.

It is this capacity to generate emergent challenges that no component can address independently that distinguishes PP 4.0 from a simple aggregation of four existing research programs. The sections that follow detail each revolution, its theoretical foundations, and how the methodological architecture proposed subsequently is designed to navigate these tensions rather than merely acknowledge them.

### Revolution 1: the regenerative revolution (from extraction to restoration)

The regenerative revolution has reconceptualized the science of wellbeing’s basic unit of analysis: moving its focus from individual optimization to systemic regeneration (Steger, 2025). Traditional approaches in positive psychology, even in its third wave, implicitly operated from within what Steger (2025) terms the “Harvest Happiness” paradigm. This extractive model argues that wellbeing is harvested from one’s psychological resources without having to attend to or address the depletion of systems that are sustaining those resources (Steger, 2025). Individuals cultivate meaning through a deep connection with nature whilst the ecological collapse keeps on accelerating (Pritchard and Richardson, 2022). People derive their purpose from making contributions to society, while social institutions around them are disintegrating (Ryff, 2024). Practitioners promote resilience while inequality deepens in their communities. Communities pursue happiness while the biosphere destabilizes. This disconnection between individual flourishing and systemic dysfunction is not a methodological problem but is rather a fundamental error in how we conceptualize and approach wellbeing.

Systemic approaches to wellbeing are not entirely new within positive psychology. Systems-informed positive psychology has been articulated as a research program since at least 2019 (Kern et al., 2020), and network approaches to psychopathology have demonstrated the value of viewing psychological phenomena as emergent properties of complex systems rather than discrete entities (Borsboom, 2017). What the regenerative revolution adds to this existing foundation is not the systemic lens per se but a normative reorientation of its purpose. Steger (2025) argues that positive psychology should undergo a fundamental reorientation in how it views human happiness by placing the focus on protecting and expanding the life-sustaining systems necessary for people to flourish. This represents a shift from “sustainability,” which seeks to maintain equilibrium within a system, to “regeneration,” which actively seeks to restore and heal the damaged ecosystems in which people live (Steger, 2025). Regenerative Positive Psychology (RPP) therefore redefines the basic unit of analysis from individual wellbeing to the health and capacity of life-sustaining systems, with the understanding that individual wellbeing naturally improves when these broader systems are flourishing (Kern et al., 2020). But the ontological status of the individual within this framework requires clarification. The present framework does not propose abandoning individual-level analysis. Even when life-sustaining systems are treated as the primary unit of concern, their assessment continues to rely substantially on individual-level data, and individual experience remains the locus of meaning-making and suffering. What changes is the interpretive frame: individual wellbeing is understood as fundamentally embedded within, dependent upon, and contributing to systemic health, rather than as a freestanding phenomenon amenable to context-free optimization.

This RPP approach mandates the field to move beyond individualized focused concepts like gratitude or nature connectedness as mere mechanisms for personal benefit, instead re-centering them on the preservation and restoration of the external environment itself. Wellbeing, in this view, depends on a network of complex systemic factors that are embedded in ecological- and sometimes non-human systems (Kern et al., 2020) which requires new interdisciplinary and post-disciplinary approaches to explain how they affect human flourishing (Wissing, 2022).

#### Expanding virtue ethics: systemic virtues

But this shift also requires us to expand the field’s view of one of the fundamental building blocks of positive psychology: strengths and virtues. It demands an expansion of the virtue ethical frameworks underpinning psychology through adopting more systems thinking approaches. In other words, we require a new framework for what is here termed *Systemic Virtues*: collective character strengths and capacities that cannot be meaningfully possessed by isolated individuals, that require multi-actor coordination to exist, that demonstrably shape or affect system-level outcomes, and that are normatively oriented toward regenerative stewardship of social and ecological systems (Van Zyl, 2026c). More specifically, they are collective character strengths and capacities that cannot be meaningfully possessed by isolated individuals, they require multi-actor coordination to exist, they need to demonstrably shape or affect system-level outcomes, and they are normatively oriented toward regenerative stewardship of social and ecological systems (Van Zyl, 2026a). These virtues are rooted in eudaimonic principles where fulfillment comes through moral virtue and purpose that transcends the self (Steger, 2025). Ecological virtues shift psychological dimensions of action away from immediate self-interest toward more informed decision-making processes where is taken responsibility for our impact on the broader system in which we live (Van Zyl, 2026c).

What distinguishes systemic virtues from traditional character strengths is their inherently relational and ecological nature (Van Zyl, 2026c). Where conventional virtue ethics often focuses on the development of individual moral character in isolation (Van Zyl et al., 2024), systemic virtues recognize that human flourishing is fundamentally interdependent with the flourishing of the broader living systems people inhabit (Poelina et al., 2022). Steger (2025) RPP emphasizes that we cannot achieve authentic wellbeing by extracting value from our (social or ecological) environments alone but rather through participating in relationships that regenerate and restore the health of these systems. Systemic virtues therefore represent capacities for what we might call an “ecological attunement.” This refers to the ability to perceive oneself as embedded within nested systems of relationships, to sense how our actions ripple through these webs of interdependence, and to cultivate patterns of behavior that enhance rather than diminish the vitality of the whole (Poelina et al., 2022). In other words, it means developing sensitivity to how our choices affect the health of ecosystems, communities, and future generations, and then acting in ways that restore rather than deplete these systems (Steger, 2025).

Traditional approaches in psychology have almost exclusively focused on understanding strengths and wellbeing from the individual’s perspective, but systemic virtues recognize that we cannot truly flourish while degrading the natural world and social fabric that sustains us. Human wellbeing and the wellbeing of the planet are therefore inseparable (Steger, 2025). The concept of systemic virtues requires stronger philosophical justification than we provide here (Van Zyl, 2026c). The question of whether relationality necessitates redefining virtues as collective properties, rather than as competencies of individuals embedded in systems, remains open (Snow, 2010). A full account would need to specify the conditions under which individual virtue instantiations are insufficient and collective-level properties become genuinely emergent (Van Zyl, 2026c). Systemic virtues are offered here as a provisional conceptual tool that warrants further philosophical and empirical development.

Further, systemic virtues also challenge the temporal boundaries of traditional ethics by extending moral consideration across space, time and generations. Regenerative thinking, as Steger (2025) articulates, requires us to shift from extractive mindsets that deplete resources for short-term gain to regenerative orientations that consider humanity’s role as ancestors to future generations and stewards of their ecological inheritance. This temporal expansion fundamentally reframes virtues like hope, courage, and wisdom by transforming them from individual psychological assets into transgenerational capacities. When we exercise systemic virtues, we engage in what might be understood as “deep time ethics” by making choices today that honor the wellbeing of beings who will inhabit these ecosystems centuries from now. In doing so, we recognize our debt to the centuries of ecological processes that made our own existence possible (Passmore et al., 2025; Steger, 2025). This represents a profound maturation of positive psychology’s understanding of purpose and meaning by moving it away from self-transcendence and more toward what we might call space–time-transcendence (i.e., recognizing that our moment-to-moment choices are threads in a much larger tapestry of planetary becoming).

A systems view of virtues, also requires a systems view of strengths or “systemic strengths.” *Systemic strengths* are collective capacities (team, organization, community, network, or society) that emerge through coordinated action and produce effects at the level of social-ecological systems (Van Zyl, 2026c). They are descriptive constructs referring to what collectives can do together that individuals cannot accomplish alone to perform functions beyond the reach of isolated individuals (Van Zyl, 2026c). These capacities arise from interaction patterns among members rather than from properties of individuals themselves, operate at collective levels spanning groups to societies, and function within nested social and ecological contexts (Van Zyl, 2026c). They are descriptive in the sense that they characterize what a system can do, given its relationships, roles, rules, and resources. Systemic strengths can be prosocial (for example, a community’s mutual aid capability) or antisocial (for example, coordinated exclusion).

This definition implies that systemic strengths are *emergent* (i.e., arising from interaction patterns between members of a collective rather than being reducible to properties of individual members themselves: Shiraev and Levy, 2024), they *operate at a collective level* and they f*unction within nested systems* (i.e., collectives themselves are embedded within larger social and ecological contexts: Kern et al., 2020; Shiraev and Levy, 2024). Systemic strengths are therefore fundamentally descriptive constructs that refers to what collectives can do together that individuals cannot accomplish alone.

Systemic strengths are different from aggregated individual traits, shared self-perceptions, and formal institutional artifacts (Meyers et al., 2023). Each of these can contribute to the emergence of systemic strengths but are in and of themselves not sufficient to be classified as collective or systemic strengths (Van Zyl, 2026c). For example, teams that are composed of highly curious individuals but may fail to develop ‘collective creativity’ because organizational norms punish dissent or disagreement. Communities championing justice and fairness may at the same time also maintain exclusionary rules. Organizations publishing sustainability reports may simultaneously increase ecological harm (Van Zyl, 2026c). None of these elements alone proves sufficient for the development of systemic strengths because systemic strengths requires an inherent alignment between individual properties, collective interaction patterns, normative structures, and actual collective functioning (Van Zyl, 2026c).

Taken together, this means new approaches must be developed for understanding collective virtues and strengths (Van Zyl, 2026c). But also, it might mean we might need to include adaptations to current conceptualizations of these to be more in line with regenerative approaches. According to Van Zyl (2026c), these could be for example:

- *Collective Creativity*: Generating innovative solutions that enhance biodiversity and ecosystem resilience rather than depleting natural capital (Van Zyl, 2026c).
- *Intergenerational Care*: Honoring ancestral wisdom while actively protecting the inheritance of future generations through present-day restoration (Van Zyl, 2026c).
- *Epistemic Humility in Complexity*: Recognizing the limits of human knowledge when intervening in living systems and maintaining epistemic openness to learning from ecosystems themselves (Van Zyl, 2026c).
- *Relational Reciprocity*: Engaging in mutual exchange with both human and more-than-human communities, ensuring that what is taken is balanced by what we give back (Van Zyl, 2026c).
- *Environmental Justice:* Building coalitions that ensure ecological burdens and benefits are distributed equitably across communities, with particular attention to those most vulnerable to environmental harm and those yet to be born (Van Zyl, 2026c).
- *Systemic Foresight:* Engaging in collaborative scenario-planning that traces how today’s choices cascade through interconnected social and ecological systems, making visible the hidden consequences of collective action (Van Zyl, 2026c).
- *Communal Sufficiency:* Co-creating community norms and practices that redefine abundance away from endless accumulation toward shared prosperity within ecological limits (Van Zyl, 2026c).
- *Ecological Stewardship*: Organizing communities of practice that pool knowledge and resources to regenerate local ecosystems, recognizing that ecological boundaries are collective thresholds requiring coordinated restraint (Van Zyl, 2026c).

These are not individual traits that happen to have collective effects but are capacities that can only exist and make sense at the collective level. In other words, you cannot practice environmental justice by yourself, and you cannot have systemic foresight without taking in multiple perspectives. Creating cultures of sufficiency require a process of shared meaning-making (Poelina et al., 2022) and ecological stewardship is by definition relational in nature (Brown and Kasser, 2005). These collective systemic virtues and systemic strengths are about what groups of people can become together, and not what isolated individuals can do on their own.

Taken together, these systemic virtues and strengths provide deeper fulfilment than fleeting material gains because they link individual action to a purpose that transcends the self (Van Zyl, 2026c) and generations (Poelina et al., 2022). They represent a fundamental expansion of Peterson and Seligman's (2004) VIA classification of character strengths to explicitly incorporate planetary stewardship as a central tenet of both individual and collective flourishing.

#### Reconceptualizing suffering as systemic feedback

This RPP also forces us to reconceptualize the field’s view of psychological suffering as also stemming from environmental degradation and social inequality. This approach views psychological suffering as stemming from dysfunctions within the macro- and meso ecosystems in which people live rather than just pathology (Comtesse et al., 2021; Cunsolo and Ellis, 2018). When people experience distress because of the destruction of the environment, this should be seen as a rational, adaptive response to real-world threats. The climate crisis is catalyzing new directions in what might be called ecological positive psychology. Researchers are investigating concepts like ecological grief (the distress experienced when witnessing environmental losses) and solastalgia (the pain of environmental change in one’s home territory) as factors that drive meaning making (Albrecht et al., 2007; Cunsolo and Ellis, 2018). This reconceptualization resonates with earlier developments in clinical psychology, particularly the network approach to psychopathology (Borsboom, 2017), which similarly challenged the view of mental health conditions as discrete latent entities and proposed instead that they emerge from dynamic interactions among symptoms within complex systems. The present contribution builds on this foundation by extending the systemic lens from psychopathology to wellbeing and from individual symptom networks to person-environment systems.

Steger (2025) regenerative positive psychology fundamentally reframes how we understand these emotional responses to ecological crisis. Rather than treating ecological grief or climate anxiety as individual pathologies requiring therapeutic intervention, regenerative approaches recognize them as appropriate psychological responses to the degradation of life-sustaining systems. This represents a critical shift from psychology’s traditional focus on individual-level adjustment toward acknowledging that distress often signals legitimate system-level dysfunction. For example, if someone experiences solastalgia it’s not because of a failure of their emotional regulation abilities but rather provides us with evidence that this person has deep relational bonds with the environment in which they live (Albrecht et al., 2007). From a regenerative perspective, the capacity to grieve for ecological losses could also be seen as something “positive.” It reflects a deep emotional connection to the living world that could motivate people towards taking more restorative actions (Comtesse et al., 2021). The challenge, however, is to ensure that this suffering is channelled in a constructive manner rather than pathologizing or suppressing it.

There is also a growing body of empirical research supporting these ideas. Research shows that these ecological emotions exist within complex psychological dynamics that can either facilitate or hinder collective action (Ogunbode et al., 2022). While climate anxiety correlates with decreased mental wellbeing across diverse national contexts, it simultaneously predicts increased pro-environmental behavior and activism (Ogunbode et al., 2022). This paradox highlights a key insight from RPP in that authentic wellbeing cannot be achieved through emotional avoidance or by optimizing individual happiness at the expense of confronting systemic dysfunctions or realities (Steger, 2025). Instead, meaning-focused coping strategies emerge as protective factors that help maintain psychological resilience whilst also sustaining engagement (Verplanken et al., 2020). These strategies involve individuals processing climate distress by connecting their emotional responses to larger purpose and collective action (Verplanken et al., 2020). Social support networks among climate activists and those sharing environmental concerns are also particularly crucial. These networks provide the relational scaffolding through which hope can be sustained, not as naive optimism, but as what researchers term “action hope”: the grounded belief that collective efforts can make a meaningful difference. This suggests that RPP interventions should cultivate communities of practice where difficult emotions can be processed collectively and transformed into sustained commitment to regenerative action.

There is also a growing body of work that examines how being connected to nature is a fundamental dimension of wellbeing (Comtesse et al., 2021). Not only is it a core component of wellbeing, but it also leads to more pro-social and pro-environmental behaviors (Comtesse et al., 2021). This creates a potentially virtuous cycle where cultivating ecological connection might simultaneously enhance individual flourishing and motivate the collective action needed to address societal and environmental crises. Some researchers explore how Indigenous relationships with land and place might inform more sustainable models of wellbeing, while others investigate how positive psychology principles can support climate activists in sustaining hope while confronting existential threats (Steger, 2025). Research is increasingly showing that nature connectedness is associated with heightened level of climate or eco anxiety (Martin et al., 2020). Paradoxically, this also correlates positively with both increased levels of psychological distress and greater pro-environmental engagement (Martin et al., 2020). This suggests that emotional responses to environmental crisis cannot be neatly categorized as either functional or dysfunctional but should rather be understood within the context in which they emerge (Martin et al., 2020).

The function of PP 4.0 interventions, through the regenerative lens, is therefore not to suppress this suffering through helping individuals develop new coping skills. But rather, it’s to help channel that suffering in constructive ways that help facilitate restorative action, necessary adaptation, and the holistic versatility required to address the physical, psychological, and philosophical changes the world needs (Wong, 2011). As Steger (2025) suggests, shifting from a “harvesting happiness” mentality toward actively restoring and expanding the vitality of the systems people inhabit requires us to honor, rather than silence, the emotional signals that alert us to ecological and systemic distress. The ultimate test of a wellbeing science is not whether it can make individuals feel better within broken systems but whether it can help heal the systems themselves (Steger, 2025).

### Revolution 2: the precision revolution (from averages to individuals)

Where the regenerative revolution examines how we are embedded within living systems, the new emerging precision revolution examines the individual definition of wellbeing and how wellbeing uniquely unfolds within each person over time. The precision revolution aims to complement the RPP transformation by ensuring that both the ideas around wellbeing (i.e., its definition, enablers and detractors) and interventions are tailored to the unique circumstances, dynamics, and meaning-making systems of each individual (Van Zyl and Dik, 2025). In other words, the precision revolution in *the science of the one*.

RPP and the precision revolution operates at two fundamentally different levels of analysis, yet both are essential for a truly comprehensive positive psychology. While regenerative approaches ask how we can restore the health of life-sustaining systems (Steger, 2025), precision approaches ask how wellbeing uniquely manifests and unfolds for each person embedded within those systems (Van Zyl and Dik, 2025). The regenerative revolution recognizes that no individual can truly flourish on a deteriorating planet or within broken social systems (Steger, 2025). The precision revolution recognizes that even within healthy systems, wellbeing is not a universal experience but rather emerges from each person’s distinctive constellation of values, strengths, vulnerabilities, contexts, and meaning-making processes (Van Zyl and Dik, 2025). One reminds us we cannot flourish on a dying planet; the other reminds us that even on a healthy planet, flourishing looks different for everyone.

This shift toward precision addresses a fundamental problem in how positive psychology has approached the study of wellbeing. Traditional positive psychology has largely operated under implicit universalist assumptions that were aimed at seeking general laws and principles that can be applied broadly across populations (Van Zyl et al., 2024). This inherently nomothetic (i.e., top-down, group-based) approach that has dominated empirical research in positive psychology (Wang et al., 2023) has helped us yield valuable insights into common features of wellbeing and how to measure/manage it at a group level (Van Zyl et al., 2024)^2^.

Van Zyl and Dik (2025) argue that positive psychology has drifted into a “science of averages” that can overlook and at times even harms the very people it hopes to help. The core issue lies in positive psychology’s implicit assumption of ergodicity (i.e., the idea that group-level patterns accurately represent individual-level experiences) (Golino et al., 2025). This occurs when, for example, researchers find that gratitude interventions lead to average changes in positive affect across their sample and they implicitly assume this finding generalizes to each unique individual within that sample (Van Zyl et al., 2024). However, psychological processes are frequently non-ergodic in nature, meaning that in many, though not necessarily all, cases. This means that patterns observed across different people at one time point do not necessarily reflect patterns within a single person across time (Golino et al., 2025). For example, a person who scores high on a measure of life satisfaction today may experience entirely different dynamics of wellbeing than someone else with that exact same score. Yet conventional nomothetic research treats these individuals and their experiences as interchangeable data points that contribute to a population average (Nissen and Beck, 2025; Van Zyl and Dik, 2025).

The consequence of this approach is not statistical in nature, because they have real world implications for how we view and approach wellbeing interventions (Van Zyl and Dik, 2025). When practitioners apply one-size-fits-all interventions based on nomothetic research, they frequently encounter individuals for whom these ‘evidence-based approaches’ simply just do not work (Nissen and Beck, 2025; Van Zyl and Dik, 2025). This is not because of poor implementation but because that person’s wellbeing dynamics differ from the average patterns on which interventions were built (Van Zyl and Dik, 2025). For example, consider two individuals who are both struggling with low levels of wellbeing. One person may lack meaning and purpose in their work which leads them to experience existential anxiety despite having strong relationships and well-developed abilities for emotional regulation. Their wellbeing plan should center on clarifying values, exploring their purpose, and restructuring work to align with what gives them personal meaning (Van Zyl et al., 2010). The other person might have ample meaning and strong relationships but suffer from emotional instability due to past trauma. Their wellbeing plan requires an entirely different focus and might include developing strategies for emotional regulation, trauma processing, and building psychological safety. Applying the same standardized intervention to both individuals ignores the unique constellation of factors driving their distress (Van Zyl and Dik, 2025). So the precision revolution proposes positioning each person’s lived experience as the foundational unit of analysis rather than treating individuals as data points within population averages.

As such, the precision revolution asks us to reconsider whether the pursuit of universal laws to wellbeing has led positive psychology astray. Perhaps the most generalizable finding in wellbeing science is that wellbeing processes may be far less generalizable than current methods assume. Perhaps the common thread connecting all human flourishing is not a shared set of ingredients but rather the deeply personal process through which each individual constructs a life that feels meaningful, authentic, and vital within their specific circumstances (Nissen and Beck, 2025; Van Zyl and Dik, 2025). This represents a shift from asking “what is the good life?” to asking “what constitutes a good life for this particular person, given who they are, where they are, and what matters most to them?” The precision revolution thus positions individual variation not as noise to be controlled but as the very phenomenon we seek to understand (Van Zyl and Dik, 2025). To be clear, the argument is not that the search for general regularities is pointless. Rather, the appropriate level of generalization must be discovered empirically through the systematic aggregation of person-specific findings, rather than assumed *a priori* through the imposition of universal frameworks. The most universal truth about human flourishing may be that it is never universal.

#### Person-specific approaches as the methodological cornerstone

Person-specific approaches, also known as N-of-1 or idiographic approaches, represent the methodological cornerstone of this precision revolution (Van Zyl and Dik, 2025). These approaches recognize that wellbeing is an inherently individual and contextually embedded experience rather than viewing it as a universal construct that manifests identically across all people (Van Zyl and Dik, 2025). In this view, wellbeing is not seen as a single experience but rather as an emergent state of positive functioning arising from the dynamic interplay between an individual’s unique inner world, including their thoughts, emotions, and motivations, their specific contexts, encompassing their environments, demands, and available resources, and their personal systems of meaning-making through which they interpret experiences (Van Zyl and Dik, 2025).

Rather than assuming universal pathways to wellbeing that apply equally to all people, person-specific approaches position the individual-in-context as the foundational unit of analysis (Nissen and Beck, 2025). These methods systematically map each person’s unique constellation of wellbeing determinants by collecting continuous streams of rich narrative, contextual, and ecological data specific to that individual over time (Nissen and Beck, 2025; Van Zyl and Dik, 2025). Through a process of collaborative meaning-making and person-centered methodologies, researchers and practitioners co-construct individualized models that capture how wellbeing unfolds and changes within that specific person’s life context and system of meaning (Van Zyl and Dik, 2025).

The distinction between nomothetic and idiographic approaches is not merely semantic but represents a fundamental shift in how we gather and interpret data about human experience (Van Zyl and Dik, 2025). Person-specific approaches require intensive repeated measurements within individuals to capture how variables relate to each other over time for that specific person. For example, an idiographic approach might examine how changes in one variable, such as feeling socially connected, correlate with changes in another variable, such as experiencing positive mood, within the same person across multiple time points. This reveals the unique psychological dynamics operating within that individual, which may differ substantially from patterns observed at the group level (Conner et al., 2009). Recent evidence suggests that the between-person trait structure identified in nomothetic research may not exist for any particular individual (Molenaar, 2004; Van Zyl and Dik, 2025). When researchers conduct person-specific factor analyses using intensive repeated measures, they find solutions ranging from two to eight factors rather than the expected five-factor structure, with individuals showing vastly different factor loadings, measurement errors, and temporal dynamics (Molenaar, 2004).

However, comprehensive person-specific approaches do not just involve extensive longitudinal data being gathered through repeated self-report measures (Nissen and Beck, 2025). Understanding what wellbeing is and how it unfolds for each particular individual requires integrating multiple streams of data that capture different facets of their experience (Van Zyl and Dik, 2025). This includes gathering objective contextual data about factors that affect their wellbeing ranging from economic conditions (e.g., GDP, inflation rate), employment opportunities in their field, housing stability, or environmental quality - all of which shape the material reality within which wellbeing must be constructed (Van Zyl and Dik, 2025). It also includes biological markers such as cortisol levels, heart rate variability, or sleep patterns which can reveal physiological stress that individuals may not consciously be aware of (Van Zyl and Dik, 2025). For example, a person might report feeling fine on a burnout questionnaire while their cortisol patterns indicate chronic physiological dysregulation which suggests that their subjective evaluation of their experience has not yet caught up with what their body is experiencing (Nissen and Beck, 2025; Van Zyl and Dik, 2025). It also includes gathering behavioral data through digital traces, wearable sensors, or ecological momentary assessments that document their actual patterns of activity, social interaction, and environmental exposure rather than relying solely on their retrospective self-report accounts (Van Zyl and Dik, 2025).

Further, person-specific approaches also capture the continuous evolution of a person’s meaning-making processes and how this affects their wellbeing over time (Nissen and Beck, 2025). The significance an individual attaches to life events, relationships, or even their circumstances is not static but shifts dynamically as they integrate new experiences and perspectives over time (Van Zyl and Dik, 2025). What one person experiences as a stressor on Monday might be reframed as an opportunity by Wednesday after a conversation with a mentor. What feels meaningful in one life stage, may feel hollow in another. Traditional assessments that treat constructs as stable entities miss this fluid interpretive process that naturally unfolds as we navigate daily life (Van Zyl and Dik, 2025). Therefore, person-specific approaches also incorporate ongoing narrative data through interviews, reflective journaling, or open-ended ecological momentary assessments that allow individuals to articulate the evolving meanings they attach to and derive from their experiences in their own words and on their own terms (Van Zyl and Dik, 2025).

Yet even with this rich tapestry of continuous streams of multimodal data, the role of the psychologist or researcher is not to impose expert interpretation of what the data means for that person’s wellbeing (Van Zyl and Dik, 2025). Instead, person-specific approaches embrace the idea of collaborative meaning-making, where practitioners and researchers partner with individuals to jointly interpret patterns in their data in order to co-construct wellbeing models that feel owned by the person (Van Zyl and Dik, 2025). The individual brings their own irreplaceable expertise about their own life context, values, and subjective experience into the conversation. The practitioner brings methodological expertise in identifying patterns and methodological tools for visualizing complex temporal dynamics. Together, they co-construct an understanding of that person’s unique wellbeing signature at that given time (Van Zyl and Dik, 2025). This process honors the individual’s agency and interpretive authority while leveraging scientific methods to reveal patterns that might otherwise remain invisible (Van Zyl and Dik, 2025). It is precisely this level of integration of multiple data streams with collaborative interpretation that makes precision approaches both scientifically rigorous and deeply personalized, paralleling the transformation that precision-sciences brought to personalized medicine in healthcare.

#### Drawing from precision medicine’s revolutionary framework

This precision revolution in positive psychology draws directly from precision medicine’s transformative methodological innovations. Precision medicine has fundamentally revolutionized healthcare by demonstrating how integrating multiple layers of biological data creates comprehensive individual profiles that enable truly personalized medical care (Ginsburg and Phillips, 2018; Manik et al., 2025). Rather than treating all patients with the same diagnosis the same, precision medicine recognizes that susceptibility to disease, progression of illnesses, and responses to treatment vary dramatically across individuals due to their unique genomic, proteomic, metabolomic, and environmental profiles (Alsaedi et al., 2025; Ginsburg and Phillips, 2018). This approach integrates genomics, which reveals an individual’s genetic makeup and inherited risk factors, transcriptomics, which captures which genes are actively being expressed, proteomics, which identifies the proteins being produced and their functional states, metabolomics, which measures the small molecules resulting from metabolic processes, and environmental data, which contextualizes how external factors interact with internal biological states (Alsaedi et al., 2025; Ginsburg and Phillips, 2018). The resulting multiomics profiles provide an unprecedented view of individual variation that has transformed clinical practice (Mani et al., 2025). For instance, two patients with seemingly identical cancer diagnoses may have entirely different molecular signatures that require fundamentally different treatment approaches (Mani et al., 2025). What appears homogeneous at the diagnostic level reveals profound heterogeneity at the molecular level (Alsaedi et al., 2025; Ginsburg and Phillips, 2018). This realization that population-level approaches are insufficient for addressing individual-level variations or needs has driven explosive growth in precision medicine approaches that prioritize individual-level data integration^3^.

What makes precision medicine’s transformation possible is the convergence of artificial intelligence and machine learning with multiomics data (Tong et al., 2023). The sheer volume and complexity of integrated biological data far exceeds human capacity for pattern recognition and interpretation (Yetgin, 2025). A single patient’s multiomics profile might include billions of data points that span genomic sequences, protein concentrations, metabolic markers, and longitudinal clinical observations that are captured through electronic health records (Yetgin, 2025). Traditional statistical approaches simply cannot handle this scale and complexity of heterogeneous data (Nam et al., 2024). Machine learning algorithms, particularly deep learning techniques, have emerged as essential tools for identifying complex, non-linear relationships within and across these massive, high-dimensional datasets (He et al., 2023; Yetgin, 2025). These algorithms can integrate data from fundamentally different domains, from molecular measurements at the cellular level to behavioral patterns captured through wearable devices, discovering patterns that would be invisible to conventional analysis (Tong et al., 2023).

Critically, AI approaches in precision medicine extend beyond simple pattern detection to enable genuine predictive modeling (Yetgin, 2025). Machine learning models trained on multiomics data can predict disease risk, treatment response, and health trajectories with unprecedented accuracy by capturing the intricate interplay between genetic predisposition and environmental exposures that unfolds uniquely within each individual (Unterhuber et al., 2021). For example, proteomics-enabled machine learning algorithms can enhance mortality prediction by identifying protein signatures specific to individual patients that conventional risk scores miss entirely (Unterhuber et al., 2021). The same computational infrastructure is now being adapted for positive psychology, where AI can process intensive longitudinal self-report data, behavioral traces from smartphones, physiological signals from wearables, and contextual information from environmental sensors to identify person-specific patterns in wellbeing dynamics (Van Zyl and Dik, 2025).

Precision medicine’s success also depends on sophisticated computational infrastructure for storing, harmonizing, and querying heterogeneous data at scale. Large-scale biobanks linked to electronic health records have created the technological foundation for integrating clinical phenotypes with molecular profiles across thousands or millions of individuals (Glicksberg et al., 2018). Projects like the UK Biobank, the U.S. All of Us Research Program, and the Electronic Medical Records and Genomics network have demonstrated that it is feasible to build secure, standardized platforms that allow researchers to conduct complex analyses across diverse data types while protecting participant privacy (Paskal et al., 2018). These platforms employ modular, extensible architectures that can integrate with existing health information systems, scale to accommodate growing data volumes, and support advanced analytics including AI model inference and data mining (Brancato et al., 2024).

The technical challenges are substantial. Integrating genomic data stored in FASTQ format with volumetric imaging data and time-series clinical observations from electronic health records requires custom interfaces, sophisticated data harmonization protocols, and robust computational infrastructure that can handle both storage demands and retrieval speeds (Brancato et al., 2024). Semantic data integration approaches using standardized ontologies help bridge heterogeneity across multiple data sources and domains, ensuring that information can be meaningfully linked despite originating from different measurement systems and clinical contexts (Prosperi et al., 2018). Positive psychology is now beginning to build analogous infrastructure (Van Zyl, 2025). Digital platforms that can integrate ecological momentary assessments, wearable sensor data, behavioral traces from smartphones, narrative responses to open-ended prompts, and contextual information about environmental and social conditions are making person-specific wellbeing research feasible at scale (Van Zyl and Dik, 2025). Cloud computing resources and distributed data architectures mean that the computational barriers that once made idiographic research impractical are rapidly dissolving.

#### From medicine to wellbeing science

The parallel between precision medicine and precision positive psychology extends beyond methodology and statistics to philosophy and into the very understanding of what wellbeing actually means. Just as precision medicine recognizes that effective treatment requires understanding the unique biological signature of each patient’s disease rather than applying population-averaged protocols, precision positive psychology recognizes that effective interventions require understanding the unique dynamics of each person’s wellbeing rather than applying one-size-fits-all programs (Van Zyl and Dik, 2025). The technological innovations that enable precision medicine, particularly AI-driven analysis of multimodal longitudinal data and computational infrastructure for secure data integration, provide both the practical tools and the proof of concept that person-specific approaches can work at scale. Where precision medicine integrates genomics, proteomics, and metabolomics to create comprehensive health profiles, precision positive psychology integrates self-report assessments, behavioral data, physiological markers, and contextual information to create comprehensive wellbeing phenotypes (Van Zyl and Dik, 2025). The transformation happening in medicine offers a roadmap for positive psychology to move beyond the science of averages toward genuine understanding of how flourishing unfolds within each unique human life.

These parallels are already materializing in empirical psychological research. Machine learning algorithms are now being deployed to predict which individuals will benefit most from which specific kinds of psychology interventions (Gyorda et al., 2023). For example, researchers have successfully used machine learning models to predict individual responses to digitally delivered worry postponement interventions by analyzing patterns in participants’ baseline characteristics and early intervention engagement (Gyorda et al., 2023). This represents a fundamental shift toward precision-guided interventions where treatment selection is informed by computational models trained on person-specific data rather than population norms (cf. Fan et al., 2025). Similarly, there is a significant rise in machine learning approaches aimed at predicting mental health trajectories among university students by integrating sociological factors, academic performance data, and psychological assessments, enabling personalized intervention recommendations tailored to each student’s unique risk profile (Nabil et al., 2022). These applications demonstrate that the AI techniques revolutionizing precision medicine can be successfully adapted to positive psychology thus creating data-driven pathways to personalized wellbeing interventions (Van Zyl and Dik, 2025).

The integration of wearable sensors with ecological momentary assessment is creating unprecedented opportunities to construct comprehensive, dynamic wellbeing phenotypes that capture how flourishing unfolds in real-time within naturalistic contexts (Henry et al., 2024). Rather than relying solely on retrospective self-reports that are collected at discrete timepoints, researchers can now combine smartphone-delivered surveys with continuous physiological monitoring from wearable devices to track the momentary fluctuations in wellbeing alongside their biological and contextual correlates (Rizvi et al., 2024). For instance, studies employing wearable biosensors measuring heart rate variability, sleep quality, and physical activity alongside repeated ecological momentary assessments of mood, stress, and social connection are revealing person-specific patterns in how these variables dynamically interact within individuals over time (Brannon et al., 2016). This multimodal data creates what might be termed “precision wellbeing phenotypes” that capture not just static traits but the temporal dynamics through which wellbeing emerges from the ongoing interplay between physiology, behavior, cognition, and context. These rich phenotypes parallel the multiomics profiles used in precision medicine, providing the empirical foundation for truly personalized interventions (Van Zyl, 2026d).

Perhaps most importantly, these technological capabilities are enabling just-in-time adaptive interventions that deliver hyper-personalized positive psychological interventions precisely when individuals need them most (Schreuder et al., 2025; von Lützow et al., 2025). By continuously monitoring patterns in sensor data and self-reported assessments, algorithms can detect early warning signs of declining wellbeing, identify high-risk moments, or recognize opportune windows for intervention, then automatically trigger context-specific support tailored to that individual’s current state (Nahum-Shani and Murphy, 2025). For example, when wearable sensors detect physiological indicators of stress combined with location data showing a person entering an environment they have previously associated with anxiety, the system can prompt evidence-based coping strategies customized to that specific situation and person (Nahum-Shani and Yoon, 2024). These just-in-time adaptive interventions represent a radical departure from traditional positive psychology programs delivered at fixed intervals regardless of individual need (Schreuder et al., 2025; von Lützow et al., 2025). Instead, they create continuous feedback loops where assessment, prediction, and intervention occur in an integrated, dynamically responsive system (Schreuder et al., 2025; von Lützow et al., 2025).

**Figure 2:**
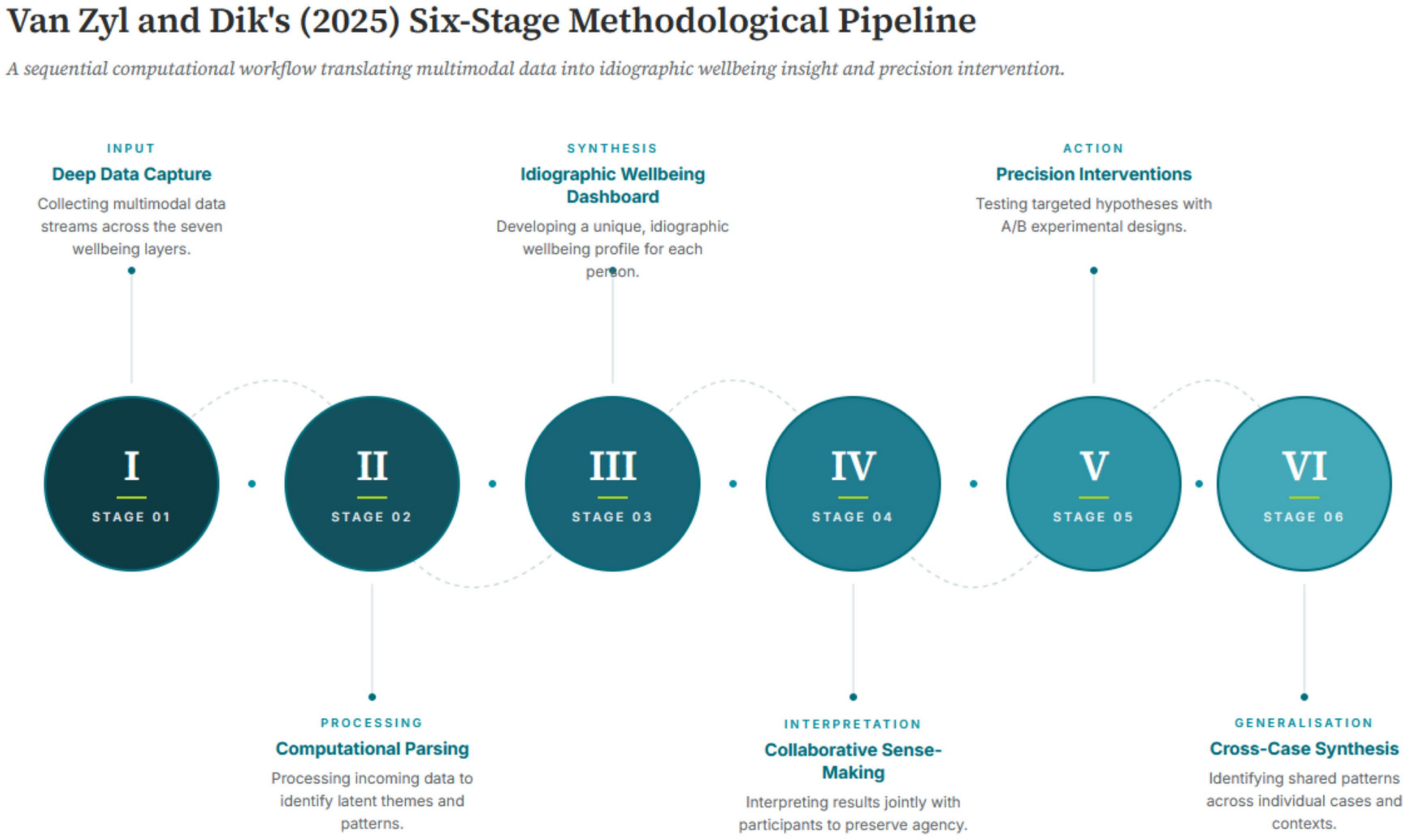
Van Zyl and Dik’s (2025) six-stage methodological pipeline.

Yet realizing this vision of a precision positive psychology demands more than just simply importing technological capabilities and innovations from medicine. It requires us to fundamentally rethink the entire research and intervention pipeline. Van Zyl and Dik (2025) outline a six-stage methodological pipeline that could act as a starting point for operationalizing this transformation (cf. Figure 2). This pipeline admittedly has a normative and programmatic character. It has not yet been empirically validated as a complete system, and its feasibility at scale remains to be demonstrated. It is presented as a structured research agenda and starting architecture rather than as established scientific practice, recognizing that each stage requires further methodological development and empirical testing.

The first stage involves deep data capture by collecting multimodal data from both subjective and objective sources (Van Zyl and Dik, 2025). This includes capturing individuals’ life stories through episodic narrative interviews, daily diaries or audio blogs, and ecological momentary assessments. These subjective data are complemented by continuous psychophysiological and behavioral measures such as heart rate variability, geolocation, and smartphone activity. Importantly, objective data can also be derived from publicly available datasets including neighborhood-level metrics like crime prevalence, air quality indices, unemployment rates, socioeconomic factors like inflation, and regional factors like safety and security. By fusing articulated meaning with tacit physiological and contextual signatures, researchers obtain a holistic perspective of each person and their environment.

The second stage applies computational parsing, where natural language techniques like topic modelling and sentiment detection analyze personal narratives while Bayesian vector autoregression models time-series data to identify recurring thematic clusters and lag-specific directional relations among variables, revealing candidate leverage points for change (Mishra et al., 2025). The objective is to identify recurring thematic clusters and estimate lag-specific directional relations among variables within the same individual, revealing candidate leverage points for change.

The third stage creates an idiographic wellbeing dashboard where researchers translate analytic outputs into an interactive network graph representing the individual’s unique wellbeing profile (Mishra et al., 2025). Nodes correspond to person-specific demands, resources, and strengths, while edges encode empirically derived influence parameters (Van Zyl and Dik, 2025). The dashboard functions simultaneously as an analytic visualization and a communicative device that renders complex dynamics in a format both researcher and participant can understand.

In the fourth stage, collaborative sense-making, researchers invite participants to engage with, provide input on, and refine their personal dashboards through guided, AI-assisted dialogue (Van Zyl and Dik, 2025). Latent wellbeing constructs are relabeled in personally meaningful language, irregular links between nodes are questioned, and priority targets for intervention are selected. This participatory process treats participants as epistemic partners rather than passive data sources, enhancing both ecological validity and fulfilling a participatory ethic (Wissing, 2022).

Once specific development targets are agreed upon, the fifth stage introduces precision interventions implemented through N-of-1 experimental designs (Van Zyl and Dik, 2025). Hyper-personalized intervention content is generated to leverage and address the individual’s unique strengths, challenges, and drivers of wellbeing (Van Zyl and Rothmann, 2019). Researchers implement strategies like alternating-treatment or A-B protocols to test hypotheses and determine the causal role of specific levers. For example, whether a brief nature micro-break can reduce the impact of midday workload spikes on evening rumination can be examined for a particular participant (Van Zyl and Rothmann, 2019).

The final stage involves cross-case synthesis, which aggregates idiographic wellbeing profiles through a mixed-methods meta-analytic process to determine overarching patterns across individuals (Kaine and Stronge, 2024). Machine learning techniques structurally cluster similar networks around similar themes, allowing researchers to develop meso-level wellbeing typologies or profiles such as high-agency creatives, collectivist caregivers, or rhythm-sensitive stabilizers (Mishra et al., 2025). These typologies act as seeds for developing new context-sensitive theories about wellbeing, achieving generalization not by averaging away diversity but by abstracting regularities that emerge across culturally and situationally heterogeneous lives.

However, this six-stage pipeline raises profound questions about the appropriate relationship between human judgment and artificial intelligence. Who ultimately decides what patterns are meaningful? How much weight should be given to algorithmic predictions versus clinical intuition or lived experience? When should AI recommendations be followed, questioned, or overridden? What happens when computational models identify patterns that conflict with a person’s self-understanding? These are not merely technical questions about implementation but fundamental issues about agency, authority, and the nature of psychological knowledge. The precision revolution demonstrates what is possible when we move beyond population averages, but it simultaneously reveals the critical importance of establishing principled frameworks for how humans and AI should collaborate in the deeply personal work of cultivating wellbeing. This recognition sets the stage for the third revolution transforming positive psychology.

### Revolution 3: the computational revolution (human-AI collaboration)

Perhaps the most transformative revolution driving PP 4.0 is the strategic integration of artificial intelligence and computational methods as fundamental element of both the way in which we understand and cultivate wellbeing. This is not simply about using technology as a tool but represents a paradigmatic shift in how positive psychology research is conducted and how interventions are delivered. The computational revolution is driving a reconceptualization of the relationship between human expertise and machine intelligence. This revolution is moving away from a model of where artificial intelligence is seen as a tool that provides technological assistance to one where there is a genuine cognitive partnership between man and machine (Sharma et al., 2023). However, PP 4.0 explicitly rejects technological solutionism, instead positioning AI within rigorous ethical frameworks centered on Human-Computer Collaboration (HCC) and Human-in-the-Loop (HITL) models. In essence, this computational revolution must be governed by rigorous ethical frameworks that position AI as augmenting rather than replacing human wisdom and judgment.

Many of the approaches discussed in this section, including digital phenotyping, machine learning in mental health, and adaptive interventions, are already well-established research programs rather than novel proposals. Concepts such as symbiotic agency (Neff and Nagy, 2016) have previously articulated frameworks for human-technology collaboration. The computational revolution’s contribution lies not in inventing these approaches but in their systematic integration into a coherent vision for positive psychology and in articulating how they interact with the regenerative, precision, and ethical dimensions of PP 4.0.

#### AI’s capabilities for advancing wellbeing science

AI systems can process large quantities and continuous streams of heterogeneous data (speech patterns, behavioral analytics, physiological responses) which offers a more comprehensive, granular and ecologically valid way of understanding wellbeing (Thakkar et al., 2024). Machine learning algorithms can recognize subtle patterns in multimodal data that are often imperceptible to human observers, enhancing both diagnostic precision and the capacity for tailored, personalized interventions (Dwyer et al., 2018; Graham et al., 2019). Recent meta-analytic evidence shows that AI-conversational agents are effective in reducing common mental health problems in the short term, with effects comparable to those of traditional face-to-face therapy (Zhong et al., 2024). These AI-conversational agents can interpret emotional cues across multiple modalities and adapt their responses to provide empathetic support (Zhong et al., 2024). They are also able to improve accessibility to care, reduce intervention costs, and provide 24/7 support that traditional mental health services cannot match (Bendig et al., 2019). These capabilities can help enable the personalization of wellbeing approaches in five principal ways which were unthinkable before.

First, AI *enhances diagnostic precision and helps with the early detection of mental health symptoms* through machine learning algorithms that can recognize subtle, complex, and non-linear patterns in human behavior that are often missed by traditional clinical assessments (Dwyer et al., 2018; Graham et al., 2019; Thakkar et al., 2024). AI can detect subtle behavioral shifts (such as sudden transitions from high to low activity) that correlate with mental health conditions like depression which helps create early warning systems for intervention. Digital phenotyping enables the detection of these subtle wellbeing changes through continuous monitoring, allowing for preventive rather than reactive approaches (Rohani et al., 2018). Studies demonstrate that machine learning models can predict depressive episodes with up to a 87% level of accuracy by only using smartphone sensor data (e.g., GPS mobility patterns, communication frequency, sleep–wake rhythms, and app usage patterns) (Farhan et al., 2016; Ware et al., 2020)^4^.

More sophisticated multimodal approaches that integrate behavioral data with linguistic markers, vocal prosody, and physiological signals achieve even higher predictive accuracies. For instance, AI models analyzing speech patterns can detect depression with up to 93% accuracy by identifying acoustic features such as reduced pitch variability, longer pauses, and slower speech rate (Low et al., 2020). Recent research demonstrates that AI can detect subtle behavioral shifts like sudden transitions from high to low activity, social withdrawal patterns, or disrupted circadian rhythms which are all associated with the emergence of common mental health problems up to 14 days before clinical symptoms become evident (Jacobson and Chung, 2020; Torous et al., 2021). This creates opportunities for positive psychology to develop early warning systems that enable preventive rather than reactive approaches which shifts the temporal frame of intervention from a crisis response to proactive wellbeing cultivation exercise.

Second, *predictive personalization in interventions* which has long been only a theoretical idea, can finally become a feasible and scalable reality. Machine learning algorithms can predict individual treatment response to positive psychology interventions, enabling precision matching of individuals to interventions based on baseline characteristics, past response patterns, and contextual factors (Collins et al., 2024). Studies demonstrate moderate correlations between baseline features (such as hope, sleep quality, self-view) and long-term outcomes at 6-month follow-up which presents evidence for the feasibility of predicting who will benefit from specific interventions (Parks and Szanto, 2013).

Advances in treatment selection algorithms now enable clinicians to estimate expected benefit from different intervention approaches with greater precision than clinical judgment alone. For example, personalized advantage index (PAI) approaches which use machine learning to estimate differential treatment benefit can improve response rates by 15–25% compared to random assignment to treatment (Kessler et al., 2016; van Bronswijk et al., 2021). In the context of positive psychology, this means we can move beyond the “one-size-fits-all” delivery of gratitude interventions, strengths-based approaches, or meaning-making exercises toward truly personalized wellbeing prescriptions that optimize intervention-person fit based on empirical prediction rather than clinical intuition alone.

Third, real-time adaptive interventions become possible through AI’s capacity for continuous learning, contextual sensing, and dynamic adjustment. Rather than static programs delivered uniformly to all participants, AI enables continuously adaptive interventions that adjust based on how each individual responds (Nahum-Shani et al., 2018; Nahum-Shani and Murphy, 2025). Smartphone apps can deliver micro-interventions throughout the day, timed to moments of need detected through passive sensing (Hekler et al., 2016). For instance, AI systems can detect patterns of stress based on heart rate variability, movement patterns, and contextual cues (e.g., being in a work location during late hours), and deliver brief mindfulness exercises, gratitude prompts, or strength-based reminders at optimal moments. Studies demonstrate that such just-in-time adaptive interventions produce significantly larger effects (*d* = 0.51–0.82) compared to time-fixed interventions (*d* = 0.28–0.34), because they deliver support when individuals are most receptive and when the need is most acute (Goldberg et al., 2022; Nahum-Shani et al., 2018). Moreover, reinforcement learning algorithms can optimize intervention timing and content over time by learning which strategies work best for each individual in different contexts, creating truly personalized intervention ecosystems that evolve with the person (Van Zyl, in press).

Fourth, *augmented therapeutic interaction* emerges through AI-driven interfaces that can interpret user emotional cues across multiple modalities and adapt their responses to provide empathetic and contextually appropriate support (D'mello and Kory, 2015; Tao and Tan, 2005). Emotionally attuned AI systems use multimodal data like facial expressions, voice prosody, physiological signals from wearables, and text analysis to detect, predict, and support emotional regulation in real-time (D'mello and Kory, 2015; Thakkar et al., 2024). Recent advances in large language models have dramatically enhanced the natural language capabilities of AI conversational agents, enabling more nuanced, contextually appropriate, and therapeutically sophisticated interactions (Sharma et al., 2023). Further, research on human-AI collaboration in mental health contexts reveals that AI-augmented peer supporters provide significantly more empathetic responses than those without AI assistance, with improvements in both cognitive empathy (understanding) and affective empathy (emotional resonance) (Sharma et al., 2023). Studies using natural language processing to analyze therapeutic conversations demonstrate that AI can identify markers of therapeutic alliance, detect ruptures in the working relationship, and suggest repair strategies that augment rather than replace clinician expertise (Ewbank et al., 2020; Imel et al., 2017). Furthermore, AI can provide real-time supervision and feedback to trainees and practitioners, identifying moments where interventions deviated from evidence-based protocols or where therapeutic opportunities were missed (Tanana et al., 2016).

Finally, AI *enables population-level wellbeing monitoring and public mental health surveillance*, creating opportunities for systemic intervention that complement individual-level approaches. Natural language processing of aggregated (and appropriately anonymized) social media data, search patterns, and digital traces can detect shifts in wellbeing, collective emotional states, emerging mental health crises, and population-level risk factors with remarkable temporal resolution (Eichstaedt et al., 2018; Low et al., 2020). In the context of positive psychology, these capabilities enable us to monitor population flourishing, detect communities experiencing collective wellbeing declines, and identify contextual factors (policy changes, economic shifts, environmental conditions) that influence wellbeing at scale. AI systems can identify communities with systematically lower access to resources that support flourishing which enables targeted public health interventions and resource allocation (VanderWeele, 2017). This population-level monitoring capacity represents a crucial bridge between individual wellbeing cultivation and the systemic, ecological approaches that PP 4.0 champions.

#### The human-in-the-loop imperative

Despite AI’s potential, it fundamentally lacks human consciousness, their embodied experience, their existential understanding, and their capacity for genuine moral reasoning (Van Zyl, 2025). As such, AI chatbots do not possess human intelligence in any comprehensive sense but rather exhibits narrow, specialized competencies within constrained domains (Chollet, 2019). PP 4.0 mandates that AI must function as a supplement to enhance care, never as replacement for human connection and judgment (Byrnes and Robinson, 2025). Psychologists must establish clear human intervention points within AI-driven workflows to maintain professional accountability (Byrnes and Robinson, 2025). The Human-in-the-Loop (HITL) imperative requires that positive psychologists establish clear human intervention points within AI-driven workflows to maintain professional accountability, ensure ethical practice, and preserve the irreducibly human elements of therapeutic work (Cai et al., 2019; Mosqueira-Rey et al., 2023).

HITL models position human experts at critical decision points in AI-mediated processes: defining objectives and values that AI systems pursue, reviewing and validating AI-generated insights before clinical application, overriding AI recommendations when contextual factors warrant alternative approaches, and maintaining ultimate responsibility for client welfare (Mosqueira-Rey et al., 2023). Research demonstrates that hybrid human-AI systems consistently outperform either humans or AI alone across diverse clinical prediction tasks, with improvements in diagnostic accuracy ranging from 12 to 37% compared to unaided clinical judgment (Gaube et al., 2021; Rajpurkar et al., 2022). However, these benefits only materialize when humans remain actively engaged in the decision-making process rather than passively deferring to algorithmic recommendations (Goddard et al., 2012).

Human positive psychologists remain crucial for overseeing and interpreting AI-generated insights, bringing clinical expertise, ethical considerations, cultural competence, and nuanced understanding of individual cases that algorithms cannot replicate (cf. Byrnes and Robinson, 2025). Clinical autonomy requires that practitioners retain both the right and the responsibility to approve or reject AI-generated recommendations based on professional judgment, therapeutic relationship quality, and contextual factors that may not be captured in the model’s training data (Byrnes and Robinson, 2025; Santoni de Sio and Mecacci, 2021). This is particularly critical in positive psychology, where wellbeing goals are inherently value-laden, culturally situated, and must be collaboratively defined with clients rather than algorithmically prescribed (Wong, 2011).

The primary ethical mandate is that AI must function as a supplement to enhance patient care and should never be used as a replacement for human connection and judgment. A central concern for computational approaches is the risk of marginalizing individuals’ subjective conscious experiences in favor of objective, third-person observational data (Gallagher and Zahavi, 2021). While AI excels at pattern recognition and prediction, it lacks the capacity for genuine empathy, existential understanding, and ethical reasoning that human practitioners bring (Montemayor et al., 2022). AI cannot understand what it is like to experience meaning, suffering, growth, or transcendence as it processes symbols but does not inhabit significance.

#### Complementary abilities between humans and AI

Research on collaborative intelligence demonstrates that humans and AI exhibit complementary capabilities that together exceed either alone (Hemmer et al., 2025). Humans excel at holistic understanding, ethical reasoning, cultural sensitivity, genuine relational connection, creative improvisation, and navigating ambiguous situations where explicit rules are insufficient. Humans bring irreplaceable capacities for compassion, moral imagination, wisdom cultivated through lived experience, and the ability to recognize and respond to the unique particularity of each person and situation (Schwartz and Sharpe, 2010). AI, on the other hand, excels at processing vast data, detecting subtle patterns across high-dimensional feature spaces, maintaining consistency in routine tasks, providing continuous availability, and performing rapid computations that would take humans extensive time. AI brings complementary strengths in scalability, reproducibility, tireless vigilance, and the ability to integrate information across temporal scales (from milliseconds to years) and data modalities that exceed human cognitive capacity (Topol, 2019). PP 4.0 leverages both parties’ strengths while compensating for the other’s weaknesses.

The optimal division of labor positions AI as handling tasks requiring pattern recognition, continuous monitoring, information integration, and routine analysis, while humans focus on tasks requiring empathy, ethical deliberation, creative problem-solving, and relationship cultivation. For instance, AI might continuously monitor digital phenotyping data and flag potential wellbeing concerns, but human clinicians make the ultimate determination of whether intervention is warranted, what form it should take, and how to collaboratively discuss this with the client. AI might suggest intervention content based on predictive algorithms, but human clinicians adapt this content based on therapeutic alliance quality, client preferences, cultural context, and clinical judgment.

Conversational swarm intelligence (CSI) represents an emerging and particularly promising form of human-AI collaboration where large groups can deliberate collectively with AI agents facilitating information flow, fact-checking, synthesis of diverse perspectives, and identification of common ground (Rosenberg et al., 2022, 2023; Rosenberg, 2016). In wellbeing contexts, this might involve communities working with AI systems to co-design culturally appropriate interventions, with AI managing information from diverse sources (research literature, community wisdom, traditional practices) while humans provide contextual wisdom, ethical guidance, and lived experience. CSI enables democratic participation in wellbeing innovation at scales previously thought impossible. This could potentially address the cultural limitations that have historically constrained positive psychology’s global applicability (Lomas, 2016).

This collaborative model creates a crucial conceptual bridge to the Positive Ethics Revolution: as AI systems become more powerful and more deeply embedded in wellbeing cultivation, the ethical frameworks governing their use become correspondingly more critical. The HITL imperative is not merely a technical design principle but an ethical commitment to preserving human agency, dignity, and moral responsibility in an age of algorithmic augmentation. The question is not whether AI will transform positive psychology (it already has) but whether this transformation will amplify or undermine the core values of human flourishing that positive psychology exists to serve. The technology to understand human flourishing at unprecedented depth already exists. The wisdom to deploy it responsibly remains the binding constraint.

### Revolution 4: the positive ethics revolution (managing algorithmic Bias and risk)

The fourth revolution relates to the creation of new ethical frameworks to manage the emerging risks and biases that AI inevitably introduces into psychological practice. The integration of AI into positive psychology is not without significant risks. Research indicates that human-AI interactions can create powerful feedback loops that amplify subtle human biases, resulting in a snowball effect greater than that observed in human-human interactions (Glickman and Sharot, 2025). Because individuals are often unaware of the extent of AI’s influence on their judgments and decisions, they are more susceptible to internalizing these amplified biases without critical evaluation (Van Zyl, 2026d). Studies demonstrate that people exhibit “automation bias” (i.e., the tendency to over-rely on automated systems and discount contradictory information from other sources) which can lead to them uncritically accepting errors AI systems make, especially when these systems are presented as being “objective” or “data-driven” (Goddard et al., 2012). Since the regenerative content of PP 4.0 champions justice and equity as systemic virtues rather than individual preferences, this risk of amplifying algorithmic biases represents one of the primary ethical threats underpinning the entire fourth wave agenda.

#### Sources of algorithmic bias

These algorithmic biases can emerge from multiple sources throughout the AI development and deployment pipeline. First, biases can emerge from training data when the datasets on which models are built over-represent certain demographics (typically WEIRD populations) while under-representing others (Obermeyer et al., 2019). This leads to algorithms performing poorly for marginalized or under-represented groups (Obermeyer et al., 2019). For instance, research on smartphone-based depression prediction algorithms reveals significant performance disparities across demographic groups, with accuracy rates of 85–91% for majority populations but below 75% for minority groups (Zhong et al., 2024). This reflects systematic biases in both data collection and algorithm design (Luger, 2025).

Second, AI systems can provide biased problem formulations when these systems operationalize wellbeing in culturally narrow ways that privilege individualistic conceptions of flourishing while marginalizing collectivist, relational, or spiritually-oriented understandings (Wong, 2011). If “successful” intervention outcomes are defined solely through Western psychological constructs (e.g., life satisfaction, positive affect, self-esteem), algorithms will inevitably reproduce these cultural assumptions, potentially pathologizing non-Western ways of being in the world.

Third, biased implementation occurs when AI systems are deployed in ways that make technology accessible to privileged populations while creating barriers for marginalized groups whether it be through cost, required literacy, language availability, or technical infrastructure (Veinot et al., 2018). Digital mental health interventions show systematic disparities in uptake, engagement, and benefit across socioeconomic status, with dropout rates 2–3 times higher among lower-income participants (Torous and Keshavan, 2020; Veinot et al., 2018).

Finally, biased interpretation occurs when clinicians, researchers, or systems uncritically accept algorithmic outputs without considering contextual factors, alternative explanations, or the limitations of the model (Luger, 2025). This phenomenon is exacerbated by the “black box” nature of many machine learning systems (Cohen et al., 2014; Gerke et al., 2020). The perceived objectivity of quantitative risk scores or algorithmic recommendations can create a false sense of certainty that obscures important uncertainties and value judgments embedded in the system (Selbst et al., 2019). There is also a risk of making biased interpretations or recommendations because an AI system’s advice is uncritically accepted. This highlights the need for developing and implementing ethical standards and safeguards to protect both positive psychologists and their clients.

#### Ethical standards and safeguards

To counteract these risks, PP 4.0 must establish rigorous standards for the ethical use and development of AI systems and implement safeguards to ensure effective governance and accountability. These ethical imperatives run parallel to the HITL technical requirements we discussed in Revolution 3, by demonstrating how computational and ethical considerations are inextricably intertwined in PP 4.0. There are a number of direct considerations that should be considered.

The most important of which is that there should be a clear focus on *transparency and continuous informed consent*. Clients must be clearly informed if, when, where, how, and for how long AI will be used in their care, with implications explained in comprehensible, non-technical language. This includes transparency about the algorithmic limitations of the system (what the AI can and cannot do), what data it was trained on and what data it uses to make decisions, and how decisions are generated. “Black box” systems that provide recommendations without explanation are ethically unacceptable in clinical contexts (Cohen et al., 2014; Gerke et al., 2020). The emerging field of Explainable AI (XAI) offers promising approaches for making algorithmic decision-making more interpretable. This includes attention-based visualizations showing which features that most influenced a prediction, counterfactual explanations demonstrating what would need to change to produce a different outcome, and local interpretable model-agnostic explanations that approximate black-box models with simpler, interpretable models (Molnar, 2020). However, research reveals that current XAI methods often fail to improve human decision-making and can even reduce accuracy when explanations are misleading or overly technical (Poursabzi-Sangdeh et al., 2021). True transparency requires both technical explainability but also clear communication about the values, assumptions, and limitations embedded in AI systems (Poursabzi-Sangdeh et al., 2021). Clients have a right to understand not just how an algorithm works but why it was designed with particular goals, what alternative approaches were not pursued, who funded and built the system, and what conflicts of interest might exist (Cohen et al., 2014; Gerke et al., 2020). Informed consent must be an ongoing process rather than a one-time event, particularly as AI systems learn and evolve over time (Cohen et al., 2014; Gerke et al., 2020).

There should be concerted efforts *to actively mitigate biases and systematically promote equity* throughout the AI development lifecycle (McDermid et al., 2021). Positive psychologists must strive to either use AI systems built on or build their own systems using high-quality, representative datasets that include data from underrepresented regions and populations. This requires intentional effort to ensure training data reflects the diversity of human experience rather than over-representing WEIRD populations (Van Zyl, 2026d). However, simply diversifying training data is not going to help if the underlying constructs, outcome measures, and intervention frameworks remain culturally narrow. Bias mitigation must occur at multiple levels and throughout the AI system’s life cycle (Van Zyl, 2026d). According to Obermeyer et al. (2019) these strategies include:

- data collection processes that actively seeks diverse participants,
- algorithm design that tests for and corrects disparate performance across groups,
- validation procedures that examine fairness metrics alongside accuracy, and
- deployment strategies that ensure equitable access and benefit

Fairness-aware machine learning approaches also offers technical approaches for reducing bias. This includes pre-processing methods that remove bias from training data, in-processing methods that incorporate fairness constraints during model training, and post-processing methods that adjust algorithmic outputs to satisfy fairness criteria (Mehrabi et al., 2021). However, fairness metrics themselves also encode value judgments into the process. For instance, whether to prioritize equal false-positive rates across groups, equal true-positive rates, or overall equality of outcomes all require explicit ethical deliberation rather than purely technical solutions (Mitchell et al., 2021).

Community-based participatory research (CBPR) approaches offer promising pathways for ensuring that AI development incorporates diverse perspectives from the outset (Hacker, 2013). By involving community members as co-researchers rather than merely research subjects, CBPR can help identify culturally relevant wellbeing indicators, appropriate intervention approaches, and potential harms that researchers might overlook (Hacker, 2013). This participatory approach embodies the conversational swarm intelligence principle discussed in Revolution 3 in order to help create genuine human-AI-community collaboration.

Next, robust data privacy and security standards for positive psychological practice must be developed and rigorously implemented (Watermann et al., 2025). There should be strict adherence to data privacy and security regulations (HIPAA in the US, GDPR in Europe, and equivalent frameworks globally), along with clear communication about how patient data is used, stored, shared, and protected (Ienca and Malgieri, 2022; Watermann et al., 2025). The aggregation of intensive multi-stream, multi-modal longitudinal data creates new privacy risks that traditional clinical data do not present (Watermann et al., 2025). Continuous monitoring can reveal intimate details about daily life, relationships, vulnerabilities, and behavioral patterns that individuals may not even be consciously aware of themselves (Insel, 2017). Traditional data protection approaches and practices are also not adequate for this context because: (1) de-identification is increasingly ineffective as rich behavioral data can be re-identified using machine learning even when direct identifiers are removed, (2) informed consent becomes problematic when individuals cannot reasonably anticipate what sensitive information might be inferred from seemingly innocuous data and (3) centralized data storage creates catastrophic failure points where single breaches can expose thousands of individuals’ intimate information (Rocher et al., 2019).

Critical perspectives on the political economy of data deserve more attention than we have given them here. Within frameworks of surveillance capitalism (Zuboff, 2019) and data colonialism (Couldry and Mejias, 2019), the human is not merely a beneficiary of AI-mediated wellbeing services but simultaneously a data source whose behavioral surplus is extracted, commodified, and deployed for purposes that may be orthogonal to their flourishing. Continuous wellbeing monitoring, however well-intentioned, operates within power relations that positive psychology cannot afford to ignore. Privacy, in this broader view, is not merely a technical problem of data protection but an existential condition related to autonomy, self-determination, and control over one’s own experience. The capacity to be unobserved, to have aspects of one’s life that are not captured, quantified, and analyzed, may itself be a precondition for certain forms of flourishing. PP 4.0 must therefore grapple with the possibility that intensive monitoring, even when technically privacy-preserving, may alter the phenomenology of experience in ways that undermine the very wellbeing it seeks to cultivate.

Blockchain technologies and federated learning approaches may offer solutions that enable data sharing for research while preserving individual privacy (Rieke et al., 2020). Federated learning allows AI models to be trained across decentralized data sources without raw data ever leaving individuals’ devices, addressing both privacy and security concerns (Rieke et al., 2020). Differential privacy techniques add carefully calibrated noise to datasets or model outputs, providing mathematical guarantees that individual-level information cannot be extracted while preserving population-level patterns useful for research (Dwork and Roth, 2014). Homomorphic encryption enables computation on encrypted data which allows AI systems to analyze sensitive information without ever accessing it in unencrypted form (Acar et al., 2018). However, these technical solutions must be complemented by robust governance frameworks that specify who has access to data under what circumstances, how long data is retained, what secondary uses are permitted, how data is destroyed when no longer needed, and what recourse individuals have if privacy violations occur (Nebeker et al., 2019). Privacy cannot be treated as a purely technical problem but must be understood as a fundamental ethical and human rights issue requiring ongoing vigilance.

PP 4.0 will *also focus on promoting trust and facilitating personal agency in human-AI interactions* (Van Zyl, 2026d). Positive psychologists should develop approaches that allow clients to “train” the AI model by providing feedback that shapes its recommendations, to understand and influence the decisions it makes over their care, and to opt out of algorithmic recommendations when desired. This helps foster a sense of psychological ownership that is valuable in addressing the complex human factors of interacting with intelligent technology (Van Zyl, 2026d). Research on human-AI trust reveals there might be a trust calibration challenge where people often exhibit either excessive trust (automation bias) or insufficient trust (algorithm aversion) with both extremes producing suboptimal outcomes (Dietvorst et al., 2015; Lee and See, 2004). Appropriate trust requires that positive psychologists understand both AI’s capabilities (what it can do well) and its limitations (where it fails) in order to adjust their reliance on AI contextually, and maintain a critical stance toward its outputs (Van Zyl, 2026d). One way to enhance trust and personal agency is through participatory AI design (Zytko et al., 2022). Here both positive psychologists and their clients can actively shape the AI system’s behavior through providing continuous feedback, specifying their preferences in how the system works/responds and suggest ways to improve collaboration with the system. For instance, allowing clients to specify which wellbeing goals matter most to them, which intervention approaches align with their values, and which data sources they are comfortable contributing creates a sense of partnership rather than surveillance. Studies demonstrate that when individuals have meaningful control over AI system behavior, they exhibit higher engagement, better adherence, and stronger therapeutic outcomes compared to non-participatory designs (cf. Habicht et al., 2025; Malouin-Lachance et al., 2025).

Moreover, *AI literacy becomes essential* for both positive psychologists and their clients (Van Zyl, 2026d). Both should have a basic understanding of how these systems work, what their recommendations are based on, and how to evaluate algorithmic advice critically. This is not simply a matter of technical education but of cultivating the capacity to engage thoughtfully and critically with AI systems while neither dismissing their potential nor accepting their outputs uncritically (Long and Magerko, 2020).

A further concern is the potential for continuous self-tracking and wellbeing monitoring to undermine intrinsic self-regulation and autonomous agency (Van Zyl, 2026a). While such systems may enhance precision, they may also foster overreliance on external data for self-understanding, diminished trust in one’s own subjective experience, and a form of dependency where individuals increasingly defer to algorithmic assessments of their own states (Van Zyl, 2026b). Research on the quantified self has documented how self-tracking can shift individuals from experiencing their lives to evaluating them against externally generated metrics (Lupton, 2016). PP 4.0 must develop design principles that mitigate these risks, for example by ensuring that monitoring systems enhance rather than replace self-awareness, by building in periods of deliberate technology disengagement, and by treating subjective experience as epistemically authoritative even when it diverges from sensor-derived data.

Finally, PP 4.0 will strive to close the digital divide and ensure equitable access to technologies that can help cultivate wellbeing. Enthusiasm for new technologies should be tempered by realizing that digital positive psychology can risk exacerbating already existing inequalities (Van Zyl, 2026d). Smartphone ownership, reliable internet access, digital literacy, comfort with technology, and language availability are not uniformly distributed within or across societies (Van Zyl, 2026d). In the United States alone, approximately 15% of adults lack smartphones, 27% of rural residents lack broadband access, and these disparities are dramatically more pronounced in low- and middle-income countries (Pew Research Center, 2021a, 2021b). Interventions that depend on sophisticated technology may be inaccessible to those who would benefit most from them (i.e., those from low-income populations, elderly individuals, rural communities, and those in developing nations) (Torous and Keshavan, 2020). Furthermore, the “algorithmic divide” refers to differential ability to benefit from AI systems, where those with higher education, digital sophistication, and cultural capital are better positioned to leverage algorithmic tools effectively (Gangadharan and Niklas, 2019). According to Van Dijk (2020) the digital divide operates at multiple levels: access (who has devices and connectivity), literacy (who possesses skills to use technology effectively), and design (whether technologies are created with diverse users in mind). Simply providing technology without addressing literacy and design dimensions can actually widen disparities rather than close them (van Dijk, 2020).

PP 4.0 must therefore develop low-tech alternatives and work to reduce barriers to digital access rather than assuming technological solutions are universally available. This might include: (1) hybrid models that combine digital tools with community-based human facilitation, (2) offline-capable applications that do not require continuous connectivity, (3) low-bandwidth designs that function on basic devices, (4) multilingual interfaces developed with and for diverse communities, and (5) integration with existing community infrastructure (schools, libraries, community centers, religious organizations) rather than requiring separate technological investment.

Moreover, PP 4.0 must remain conscious of the fact that technological innovations cannot solve social problems if we do not also address the underlying structural inequities that cause them (Morozov, 2013). In other words, digital mental health interventions cannot replace the mental health workforce, nor can it improve access to accessible healthcare systems. It can also not provide liveable wages, safe housing, and other social determinants that are imperative for driving wellbeing (Morozov, 2013). Therefore, technology must be positioned as one tool among many in a comprehensive approach to human flourishing and should not be seen or positioned as a replacement for the social investment in positive psychology’s wellbeing infrastructure.

#### Ethical AI as foundational to PP 4.0

The Positive Ethics Revolution is not one revolution among four. It is the foundational architecture upon which the three preceding revolutions must be built. Without robust ethical frameworks governing algorithmic systems, the regenerative virtues of justice and equity become performative rhetoric, ecological approaches risk perpetuating systemic biases at population scales, and computational capabilities amplify rather than ameliorate existing inequities. The technical innovations of an AI-augmented positive psychology, from diagnostic precision and predictive personalization to adaptive interventions and augmented therapeutic interaction, can only serve genuine human flourishing when embedded within ethical systems that prioritize transparency, fairness, privacy, agency, and equity. Ethics is not a constraint on innovation but the compass that ensures innovation serves humanity rather than subjugates it.

Recent work sharpens the urgency of this claim. The AI-IARA framework (Van Zyl, 2026a) identifies six irreducible psychological capacities essential for maintaining human agency under algorithmic conditions: Awareness (the ability to detect when AI influences cognition and decision-making), Interpretation (the capacity to generate personal meaning from experience), Intention (the ability to choose values before optimization constrains the choice set), Action (the capacity to sustain effortful, self-directed behavior), Relational Agency (the ability to maintain authentic human connection), and Autonomy (the meta-capacity to consciously calibrate AI reliance across contexts). Each of these capacities faces systematic erosion through cognitive offloading, automation bias, and attention fragmentation (Van Zyl, 2026a). Crucially, the AI-IARA framework reframes the ethical question from whether AI systems function correctly to whether they preserve or erode the psychological capacities required for genuine flourishing. This has direct implications for PP 4.0: the very technologies deployed to enhance wellbeing monitoring and intervention may simultaneously undermine the self-directed agency that authentic wellbeing requires. Designing AI systems that strengthen rather than atrophy AI-IARA capacities becomes a fundamental design requirement for any ethically defensible implementation of the fourth wave. Psychology’s most urgent task is no longer to optimize human performance but to architect the conditions under which genuine human agency remains possible.

Translating these principles into concrete guidance requires specifying what positive psychologists should do differently in their daily practice. At minimum, practitioners should: (a) develop basic competence in evaluating AI system limitations and biases before integrating them into clinical workflows, (b) establish explicit informed consent protocols that address not only data use but the potential psychological effects of AI-mediated interventions, (c) maintain regular human contact as a non-negotiable complement to any AI-assisted intervention, (d) advocate within their institutions for equitable access to technology-enhanced services, and (e) engage in continuing professional development specifically focused on the ethical dimensions of AI in psychological practice. These recommendations are provisional and will require elaboration through professional consensus-building processes.

This integration of computational innovation and ethical vigilance represents PP 4.0’s most defining characteristic: embracing transformative technologies while remaining anchored to human dignity, justice, and universal flourishing. The existential question confronting the field is whether positive psychology will actively shape this technological transformation according to its deepest ethical commitments or passively allow technological imperatives to redefine human flourishing itself. Answering this question requires not only philosophical clarity on the role of AI in wellbeing but new methodological innovations capable of rigorously evaluating these complex, technologically-mediated approaches to human experience. It is to that methodological architecture that the paper now turns.

## A methodological architecture for PP 4.0

The previous section articulated the conceptual foundations of PP 4.0’s four revolutions. However, conceptual frameworks that are not coupled with the methodological advancements to realize such will remain nothing more than just an aspirational pipedream. This section presents a comprehensive methodological architecture for PP 4.0 by detailing the specific techniques, technologies, and approaches required to operationalize its different revolutions. Drawing on parallel developments in precision medicine, digital twin technology, biosensor innovation, network neuroscience, and quantum computing, we articulate how positive psychology will conduct research and deliver interventions in the coming decade.

### Digital twins for personalized wellbeing

Digital twin technology represents one of the most promising methodological innovations for PP 4.0. A digital twin is a dynamic virtual replica of a physical entity with bidirectional links between the physical and digital domains (Vallée, 2024). In healthcare, digital twins integrate diverse data sources including genomics, proteomics, imaging, sociodemographic, and real-world behaviors to create patient-specific simulations that model disease progression, optimize treatments, and personalize interventions (De Domenico et al., 2025). PP 4.0 proposes developing Wellbeing Digital Twins: dynamic computational models of individuals that integrate multimodal data streams to predict wellbeing trajectories and optimize personalized interventions.

A Wellbeing Digital Twin is not a dashboard, a static profile, a psychometric score, or a one-time assessment report. It is a living hypothesis about a person: a continuously evolving computational model that learns from every new data point, simulates potential scenarios, maps how someone thinks, adapts, and grows, and is always current and always updating (Vallée, 2024; Zalmanovici-Meisel and Goldsmith, 2023). This is an important distinction because the field of psychology has historically relied heavily on episodic, cross-sectional snapshots of human functioning in order to infer a person’s mental state (Van Zyl et al., 2024). The digital twin paradigm fundamentally challenges this temporal logic by replacing periodic measurement with continuous modelling, reactive assessment with predictive simulation, and static categorization with dynamic representation (Barricelli et al., 2019).

Zalmanovici-Meisel and Goldsmith (2023) laid the conceptual foundations for mental health digital twins by demonstrating how a virtual representation of an individual’s mental states and processes, continually updated from data collected over the lifespan, could guide professionals in diagnosing and treating patients through mechanistic models and machine learning tools. Their framework establishes the probabilistic coupling between the physical person (whose states evolve over time) and the digital model (which tracks, predicts, and simulates those states). This bidirectional architecture is fundamental: the digital twin is not a passive record but an active model that generates predictions, tests hypotheses, and updates its parameters as new observational data stream in (Zalmanovici-Meisel and Goldsmith, 2023). Further, digital twins allow us to develop and implement hyper-personalized interventions, monitor trajectories, and affords us new ways to anticipate changes within individuals as they occur or even before symptoms present themselves (Van Zyl and Dik, 2025). This anticipatory capacity represents a qualitative shift from psychology’s traditional reactive stance.

**Figure 3:**
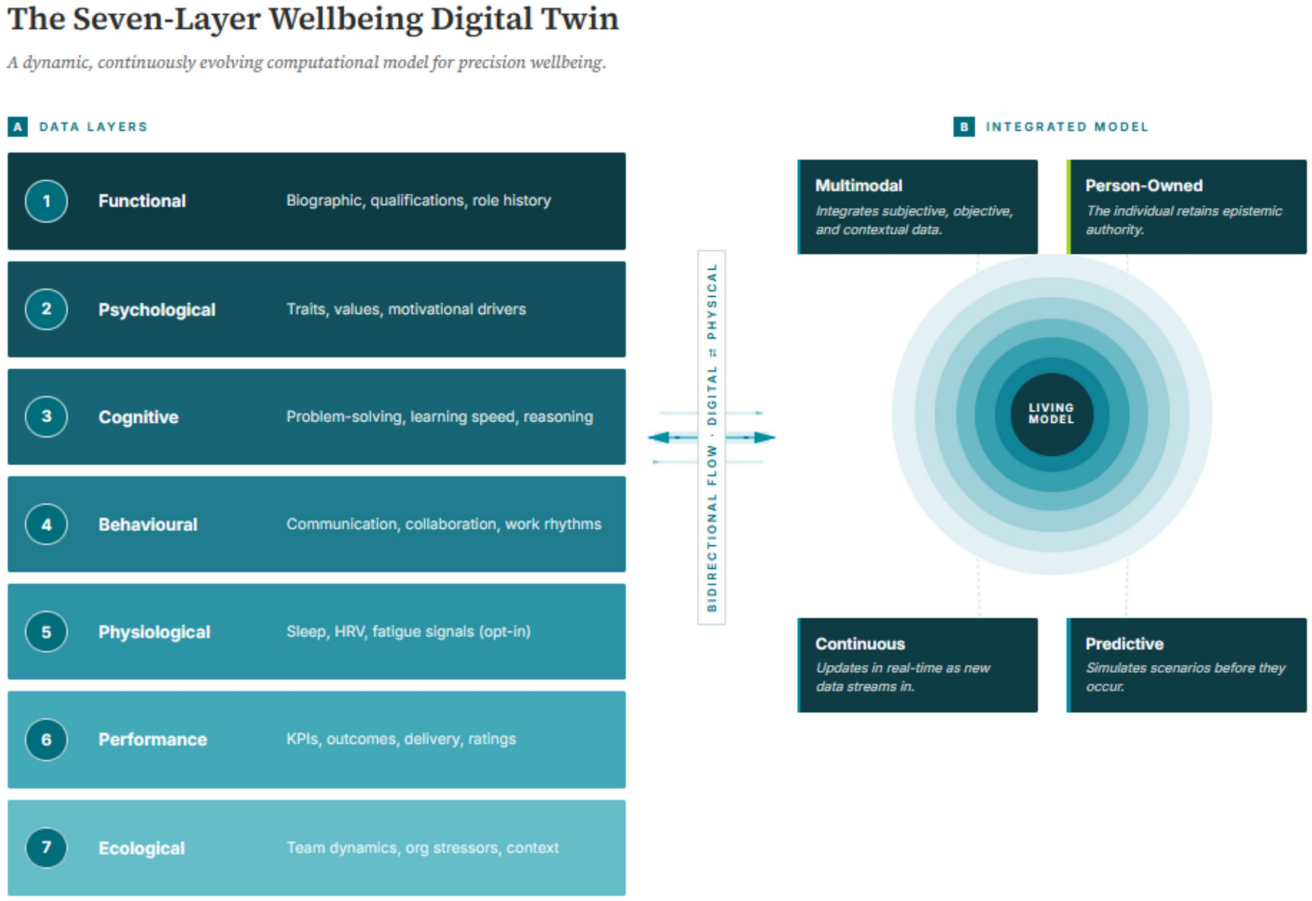
Seven layer-framework for a digital wellbeing twin.

We propose a *seven-layer architecture for the Wellbeing Digital Twin* (see Figure 3) that extends existing digital twin frameworks from precision medicine into the domain of positive psychology. Each layer contributes continuous data streams that are computationally integrated into the living model.

1. The first layer, the functional layer, captures biographic foundations and role context, including qualifications, role history, certifications, and career trajectory. These data provide the structural scaffolding within which other layers are interpreted. A person’s functional context shapes the meaning and significance of patterns observed in other layers (Van Zyl and Dik, 2025).
2. The second layer, the psychological layer, models stable traits, values, and motivational architecture, including personality dimensions, core values, motivational drivers, and stress response patterns. Unlike traditional psychometric assessment that captures these constructs at discrete timepoints, the digital twin treats them as parameters that can shift over developmental time, updating estimates as new data provide evidence of change (Molenaar, 2004; Nissen and Beck, 2025).
3. The third layer, the cognitive layer, captures information processing and adaptive reasoning, including problem-solving style, learning speed, reasoning approach, and decision quality under uncertainty. Recent advances in digital cognitive twins demonstrate that dynamic computational models of cognitive states can integrate neuropsychological assessments with real-time biometric data to provide personalized and adaptive cognitive monitoring (Barricelli et al., 2019).
4. The fourth layer, the behavioral layer, tracks observable action patterns in naturalistic contexts through digital traces, including communication patterns, collaboration style, work rhythms, and interaction dynamics. Digital phenotyping research has demonstrated that passively collected behavioral data from smartphones and wearables can reveal patterns associated with wellbeing changes that self-report measures miss entirely (Torous et al., 2021).
5. The fifth layer, the physiological layer, integrates biological signals from opt-in wearable sensors, including sleep quality, heart rate variability, cortisol patterns, fatigue signals, and activity levels. This layer draws directly on the biosensor innovations discussed earlier and represents what precision medicine terms the ‘continuous monitoring’ paradigm, where single-timepoint measurements are replaced by dense temporal streams (Duan et al., 2025; Vo and Trinh, 2024).
6. The sixth layer, the performance layer, captures outcome metrics and competence indicators, including task outcomes, quality metrics, project delivery, and peer evaluations. This layer provides the criterion data against which the predictive validity of the twin’s models can be evaluated and refined.
7. The seventh layer, the ecological layer, models the systemic context shaping individual experience, including team dynamics, organizational stressors, market conditions, and workload context. This layer operationalizes the regenerative revolution’s insistence that individual wellbeing cannot be understood apart from the systems in which it is embedded (Steger, 2025). It ensures that the digital twin does not reproduce the decontextualized individualism that PP 4.0 seeks to overcome.

Together, these seven layers generate thousands of continuous data points that are computationally integrated through the modelling infrastructure described below. The critical advantage over traditional assessment is not merely the volume of data but its temporal density and multimodal integration. Where a traditional wellbeing assessment provides a photograph, the seven-layer digital twin provides a continuously updating film (Vallée, 2025).

The most transformative capability of the Wellbeing Digital Twin is the ability to run simulations on the individual’s digital twin. With seven layers of continuously updated data, the twin enables researchers and practitioners to run computational simulations against the model before any intervention is delivered to the person. Scenario testing, such as simulating how an individual might respond to a role transition, a period of elevated workload, or a specific wellbeing intervention, can be computationally explored before real-world implementation (De Domenico et al., 2025; Vallée, 2025). This shifts the logic of assessment from retrospective evaluation to prospective prediction, and the logic of intervention from one-size-fits-all delivery to pre-tested, person-specific optimization.

However, this simulation capacity raises profound ethical questions that connect directly to the Positive Ethics Revolution. As Van Zyl (2026a) argues through the AI-IARA framework, systems that model and predict human behavior must be designed to preserve rather than erode human agency. The guiding ethical principle proposed here is this: your digital twin should never be more certain about you than you are about yourself. The twin must function as a tool for collaborative sense-making, not as an authoritative oracle that supplants self-understanding. The individual retains epistemic authority over their own experience, and the twin’s predictions must always be presented as hypotheses to be evaluated in partnership with the person, not as determinations to be imposed upon them (Van Zyl and Dik, 2025).

These models would leverage differential equations for modelling wellbeing dynamics, Bayesian networks for multiomics integration, Markov models for state transition probabilities, and reinforcement learning for treatment optimization (Vallée, 2025). Unlike static assessments capturing wellbeing at discrete timepoints, digital twins continuously update as new data streams in, enabling real-time prediction and intervention adaptation.

The feasibility of this approach is supported by developments in adjacent fields. In oncology, digital twins that integrate molecular data with clinical observations have demonstrated the capacity to predict treatment response and optimize intervention timing at the individual level (De Domenico et al., 2025). In cardiology, digital twin models have achieved clinically meaningful prediction of disease trajectories using continuous sensor data (Vallée, 2024). While psychology’s constructs present measurement challenges that differ from those in medicine, particularly regarding the reliability and validity of continuous multimodal data streams, the architectural principles transfer: bidirectional coupling, continuous updating, and predictive simulation are technically feasible and conceptually appropriate for wellbeing science.

### Continuous multimodal phenotyping

Precision positive psychology requires moving beyond periodic self-report assessments to continuous multimodal monitoring capturing wellbeing determinants in real-world settings. Recent advances in wearable biosensor technology enable this transformation. Modern biosensors integrated into clothing, accessories, or applied directly to skin provide continuous real-time monitoring of physiological and biochemical parameters including heart rate, glucose levels, cortisol, and hydration status (Vo and Trinh, 2024). Next-generation biosensors leverage microfluidic systems for non-invasive analysis of sweat, saliva, tears, and interstitial fluid, while smart tattoos, microneedle patches, and flexible bioelectronics provide minimally invasive access to critical biomarkers (Duan et al., 2025). Machine learning algorithms process biosensor data streams to identify patterns predicting wellbeing changes (Zhou et al., 2024). For instance, machine-learning-powered wearable sensors can distinguish and predict sweat biomarker patterns associated with different psychological states (Vo and Trinh, 2024).

PP 4.0 proposes developing Psychological Biosensor Panels that are analogous to the metabolic panels used in medicine. These panels would continuously monitor multiple biomarkers relevant to wellbeing including cortisol (stress response), heart rate variability (autonomic regulation), inflammatory markers (systemic physiological stress), neurotransmitter metabolites (mood regulation), and sleep architecture (recovery processes). Machine learning algorithms can process these biosensor data streams to identify patterns predicting wellbeing changes, with recent studies successfully using wearable physiological data integrated with differential privacy to recognize emotions while preserving user privacy (Benouis et al., 2024; Zhou et al., 2024). Combined with behavioral data from smartphone sensing and periodic self-reports, these panels enable constructing comprehensive wellbeing phenotypes revealing within-person patterns invisible in traditional between-person designs (Vo and Trinh, 2024).

The convergence of biosensors with smartphones enables Ecological Momentary Assessment at scale and granularity (Rizvi et al., 2024). Passive sensing captures accelerometer data, GPS location, social interaction patterns through call logs and messaging frequency, app usage, and physiological information through wearable integration. However, systematic reviews reveal that no single data source captures all aspects of wellbeing: psychological symptoms are best captured by linguistic features, physical symptoms by wearable sensors, social symptoms by communication patterns, and physiological symptoms by biosensors (Van Zyl, in press). This reality necessitates sophisticated multimodal fusion architectures that can provide a comprehensive moment-by-moment quantification of an individual’s behavioral and physiological state (Van Zyl, in press).

Recent work demonstrates the power of integrating diverse assessment and analytic modalities. Combining large language models for text analysis with facial expression recognition in video data significantly improves depression severity prediction beyond either modality alone (cf. Fleig, 2025). Virtual reality environments integrated with multimodal physiological sensing including EEG, eye-tracking, and heart rate variability offer particularly promising assessment frameworks, achieving high diagnostic accuracy for adolescent depression while providing objective, standardized environments (Garcia-Ceja et al., 2018; Wu et al., 2025). Combined with behavioral data from smartphone sensing and periodic self-reports, these multimodal approaches enable constructing comprehensive wellbeing phenotypes revealing within-person patterns invisible in traditional between-person designs (cf. Wu et al., 2025).

### Network neuroscience and brain-behavior mapping

Traditional positive psychology treats brain regions as isolated units performing discrete functions. Network neuroscience demonstrates this localizationist approach is fundamentally misguided (Barabási et al., 2023). Brain function emerges from complex interactions among distributed networks, with wellbeing-related processes involving coordinated activity across multiple systems. PP 4.0 must embrace network approaches to understanding the neural bases of wellbeing. Functional connectivity analysis using fMRI reveals statistical dependencies between brain region time series, mapping large-scale networks supporting positive experiences (Sporns, 2017). Structural connectivity analysis using diffusion tensor imaging can trace white matter pathways that connect different brain regions (Sporns, 2017) and effective connectivity modelling can estimate causal influences between brain regions (Santoro et al., 2024). Higher-order connectomics captures group interactions beyond pairwise connections, revealing topological signatures associated with cognitive states and individual differences (Santoro et al., 2024).

Network neuroscience provides methodological tools for mapping how wellbeing emerges from brain network organization. Graph theoretical metrics quantify network properties including integration (global efficiency), segregation (modularity), centrality (hub identification), and resilience (robustness to perturbation) (cf. Artime et al., 2024). These metrics can serve as biomarkers predicting intervention response and tracking wellbeing changes. Critically, network approaches reveal that brain organization is not static but dynamically reconfigures across time and contexts (Bassett et al., 2011). Dynamic network analysis captures temporal fluctuations in connectivity, revealing how brain networks transition between functional configurations (Finc et al., 2020). PP 4.0 interventions could target network dynamics rather than static regional activations, promoting flexible reconfiguration supporting adaptive responses to environmental demands.

### Artificial intelligence and large language models

The emergence of large language models and generative AI represents a transformative development for positive psychology research and practice. These technologies are capable of analyzing natural language, generating personalized interventions, and providing scalable wellbeing support. Recent systematic reviews document explosive growth in LLM applications for mental health, with research publications increasing dramatically since 2022 (Jin et al., 2025).

Current applications span three primary domains. First, LLMs analyze linguistic patterns in social media posts, clinical interviews, and self-reports to detect mental health conditions with accuracy often exceeding traditional methods (Jin et al., 2025). Second, LLM-powered conversational agents provide cognitive-behavioral interventions, psychoeducation, and real-time support, with randomized controlled trials demonstrating effectiveness for reducing depression and anxiety symptoms (Lawrence et al., 2024). Third, LLMs accelerate literature reviews, generate hypotheses, identify patterns in large datasets, and support theory development through natural language understanding of complex psychological phenomena (Lawrence et al., 2024).

However, this enthusiasm must be tempered by critical evaluation. Studies document inconsistent performance in crisis situations. When compared with expert suicidologists using standardized suicide intervention inventories, LLMs showed upward bias in judging responses as appropriate and performance varied substantially by model (Moore et al., 2025). Various global health and technology institutions have issued detailed ethics and governance guidance, calling for transparency, rigorous evaluation, and oversight proportionate to risk (Van Zyl, 2026d). Critical challenges include hallucinations and factual inaccuracy, bias and fairness concerns, privacy and security risks, lack of true understanding, and potential for harm in crisis situations (Park et al., 2025).

PP 4.0 must adopt evidence-based, ethically grounded approaches to LLM integration. This includes rigorous testing before deployment, human oversight mechanisms through human-in-the-loop protocols, transparency about AI involvement, and clear delineation between AI augmentation of professional care versus replacement. LLMs represent powerful tools for positive psychology, but only when deployed with appropriate safeguards and realistic expectations about their capabilities and limitations.

### Quantum-enhanced pattern recognition and optimization

While quantum computing remains in its infancy, understanding its potential is essential for envisioning PP 4.0’s long-term trajectory. Unlike classical computers that process information as discrete bits, quantum computers leverage the principles of superposition and entanglement to explore multiple solution pathways simultaneously (Garg et al., 2025; Stefano, 2024). This fundamental difference means that for certain types of problems, particularly those involving pattern recognition in high-dimensional data and complex optimization, quantum approaches could eventually not only provide new insights but help analyze masses of data in a fraction of a second (Garg et al., 2025; Stefano, 2024).

For positive psychology, three potential applications merit attention over the coming decade. First, quantum machine learning algorithms may eventually identify subtle patterns in multimodal wellbeing data that remain invisible to current analytical approaches (cf. Stefano, 2024). When faced with datasets integrating biosensor readings, neuroimaging, behavioral patterns, genetic markers, and contextual information simultaneously, quantum algorithms could theoretically detect the complex, nonlinear interactions that drive individual differences in flourishing. Second, quantum optimization might solve what are currently intractable matching problems for determining which intervention approach, delivered at what intensity, through which modality, will optimize outcomes for a specific individual given their unique constellation of characteristics and constraints (Stefano, 2024). Third, quantum simulation could model wellbeing as a truly dynamic system by capturing emergent properties and feedback loops that classical models struggle to represent (Stefano, 2024).

However, at the moment quantum hardware remains unstable, error-prone, and difficult to scale beyond laboratory demonstrations (Stefano, 2024). More critically, systematic reviews find no consistent performance advantages for quantum machine learning over classical methods when evaluated under realistic operating conditions (Ullah and Garcia-Zapirain, 2024). The promise of quantum computing for wellbeing science lies not in immediate applications but in its potential to fundamentally expand what becomes computationally possible as the technology matures.

PP 4.0 must therefore adopt a watchful waiting stance. We monitor quantum developments, prepare conceptual frameworks for eventual integration, and ensure data architectures remain compatible with future quantum approaches. Yet our primary methodological investments must focus on classical computational methods that deliver demonstrable benefits today. Quantum computing represents aspirational infrastructure for the 2030s rather than immediately deployable methodology for PP 4.0’s foundational decade.

### Participatory system design and indigenous epistemologies

While advanced computational methods enable precision at individual levels, PP 4.0’s regenerative focus demands collaborative methodologies ensuring interventions are systemically appropriate and culturally valid. Participatory Action Research (PAR) becomes central here (Van Zyl, 2025). PAR seeks to understand the world by actively changing it, emphasizing participation and action by members of affected communities (Fine and Torre, 2021).

PAR is critical for the regenerative agenda because it shifts research from being imposed on communities to being conducted in partnership, founded upon community voices and agency (Fine and Torre, 2021). PAR structurally integrates participation (democracy), action (engagement), and rigorous research (soundness) (Fine and Torre, 2021). It is particularly vital for cultural adaptation of interventions, aligning with Indigenous conceptualizations of health and wellbeing, which are often holistic and strengths-based.

Indigenous positive psychologies represent frameworks developed within and by specific cultural communities rather than adapting Western models to non-Western contexts (Van Zyl et al., 2024). African Ubuntu philosophy views wellbeing as fundamentally relational (“I am because we are”: Mayisela et al., 2026). Māori concepts like whānau (extended family) and whakapapa (genealogy and connection to ancestors) position flourishing within intergenerational and spiritual contexts (Boulton and Gifford, 2014). Buddhist psychology offers sophisticated models centered on metta (loving-kindness), mudita (sympathetic joy), and equanimity that predate Western psychology by millennia (Van Zyl et al., 2024).

These represent genuinely different ontologies and epistemologies about what constitutes a good life (Wissing, 2022). PP 4.0 must honor these diverse epistemologies not merely as cultural adaptations but as fundamentally valid ways of knowing that may operate with different validity criteria than Western science traditionally demands (Van Zyl, 2025). Crucially, PAR is structurally necessary for ethically gathering high-quality, culturally informed data required to develop robust and unbiased AI models for global application.

### Person-specific experimental designs

PP 4.0 embraces Person-Specific (N-of-1) experimental designs as the methodological cornerstone of precision science approaches. These single-case randomized clinical trials are optimal for studying interventions requiring repeated, often daily application (Lillie et al., 2011). While multiple subjects are typically studied, the focus of N-of-1 designs remains establishing precise effects for each specific individual (Lillie et al., 2011). These designs employ multiple crossover periods, determining optimal wellbeing strategies for specific individuals rather than relying on between-person averages that may not apply to any particular individual (Lillie et al., 2011).

Advanced statistical methods analyze intensive longitudinal data generated by precision approaches. Group Iterative Multiple Model Estimation (GIMME) effectively combines nomothetic and idiographic advantages. GIMME creates person-specific maps showing how intensively measured variables predict each other across time scales, while simultaneously identifying common group-level structures. Vector Autoregressive (VAR) approaches robustly investigate idiographic psychological processes, modelling how variables influence each other within single person’s experience (Piccirillo and Rodebaugh, 2019).

Machine learning for pattern recognition identifies complex nonlinear relationships in multimodal data streams (Collins et al., 2024). These algorithms integrate data from wearables, self-reports, behavioral tracking, and contextual information to identify individualized wellbeing determinants (Collins et al., 2024). For instance, machine learning might reveal that for one person, wellbeing declines primarily in response to sleep disruption, while for another, social interaction quality is the dominant predictor. Van Zyl and Dik (2025) demonstrate how machine learning can develop hyper-personalized wellbeing models based on narrative data, self-reports, and objective macro-level data sources, enabling tailored interventions designed specifically for each individual’s unique drivers of flourishing.

### Privacy-preserving methodologies

As PP 4.0 embraces comprehensive data collection, protecting participant privacy becomes paramount. Federated learning has emerged as a transformative approach enabling collaborative model training while keeping sensitive data decentralized and private (Khalil et al., 2024). Federated learning operates through a client–server architecture where a centralized model is trained using data distributed across multiple clients, ensuring data remain locally and are never transmitted to central servers (Khalil et al., 2024). This approach addresses critical privacy concerns in mental health research where data are inherently personal and stigmatized (Khalil et al., 2024).

Studies show growing applications in depression detection from social media and mobile data, stress and anxiety recognition from multimodal sensors, bipolar disorder transition prediction from electronic health records, sleep behavior analysis from wearable devices, and emotional recognition from physiological signals (Vajrobol et al., 2025). Recent innovations integrate federated learning with other privacy-enhancing technologies. Differential privacy adds calibrated noise to protect individual contributions while maintaining model utility (Benouis et al., 2024). Domain-aware privacy budgets adapt privacy protections to data sensitivity, with mental health text receiving stronger guarantees. Low-rank adaptation enables efficient fine-tuning of large language models by updating only small parameter subsets, reducing communication costs and privacy risks (Sarwar, 2025).

However, federated learning faces particular challenges when data are non-independent and identically distributed across clients, common in mental health where populations differ in demographics, symptom presentations, and cultural contexts (Khalil et al., 2024). Research shows federated learning performs comparably to centralized models when data distributions are similar but faces challenges when substantial disparities exist (Khalil et al., 2024). PP 4.0 must develop frameworks robust to data heterogeneity while maintaining fairness across diverse populations. The integration of federated learning with multimodal data fusion represents a frontier, with recent frameworks demonstrating feasibility of federated multimodal models combining demographic data, physiological signals, and behavioral patterns for mental health prediction while preserving privacy (Khalil et al., 2024).

### Integrative methodological framework

The methodological advances described above are not isolated techniques but interconnected components of an integrative research architecture. PP 4.0’s methodological power emerges from synergistic combinations. Digital twins integrate continuous multimodal phenotyping with causal models and personalized predictions. Explainable AI ensures complex machine learning models remain interpretable and trustworthy. Federated learning enables large-scale collaborative research while protecting privacy. Network science reveals system-level dynamics complementing individual-level precision approaches. Participatory methods ensure technological sophistication serves genuine human flourishing across diverse cultural contexts.

This integrated architecture enables PP 4.0 to realize its conceptual vision: precision psychology that is personalized, predictive, preventive, and participatory which is conducted at scale while respecting privacy, rooted in causal understanding, and committed to regenerative rather than extractive relationships with individuals, communities, and planetary systems. The coming decade will see these methodologies mature from emerging innovations to established best practices. Success requires not only technical excellence but ethical wisdom, ensuring that increasingly powerful tools for understanding and influencing wellbeing serve human flourishing in its fullest, most culturally diverse, and most systemically regenerative expressions.

### From architecture to application: the adaptive twin-led assessment system (ATLAS)

The methodological components outlined above, digital twins, continuous multimodal data, computational parsing, and collaborative sense-making, are powerful individually but remain fragmented without an integrating architecture. The *Adaptive digital Twin-Led Assessment System* (ATLAS; Van Zyl, in press) provides one specification of what such integration looks like when applied to the domain of psychological assessment. ATLAS is a conceptual architecture that demonstrates how the four revolutions of PP 4.0 converge in a concrete methodological system, and it is introduced here because it makes the abstract principles of the preceding sections falsifiable.

**Figure 4:**
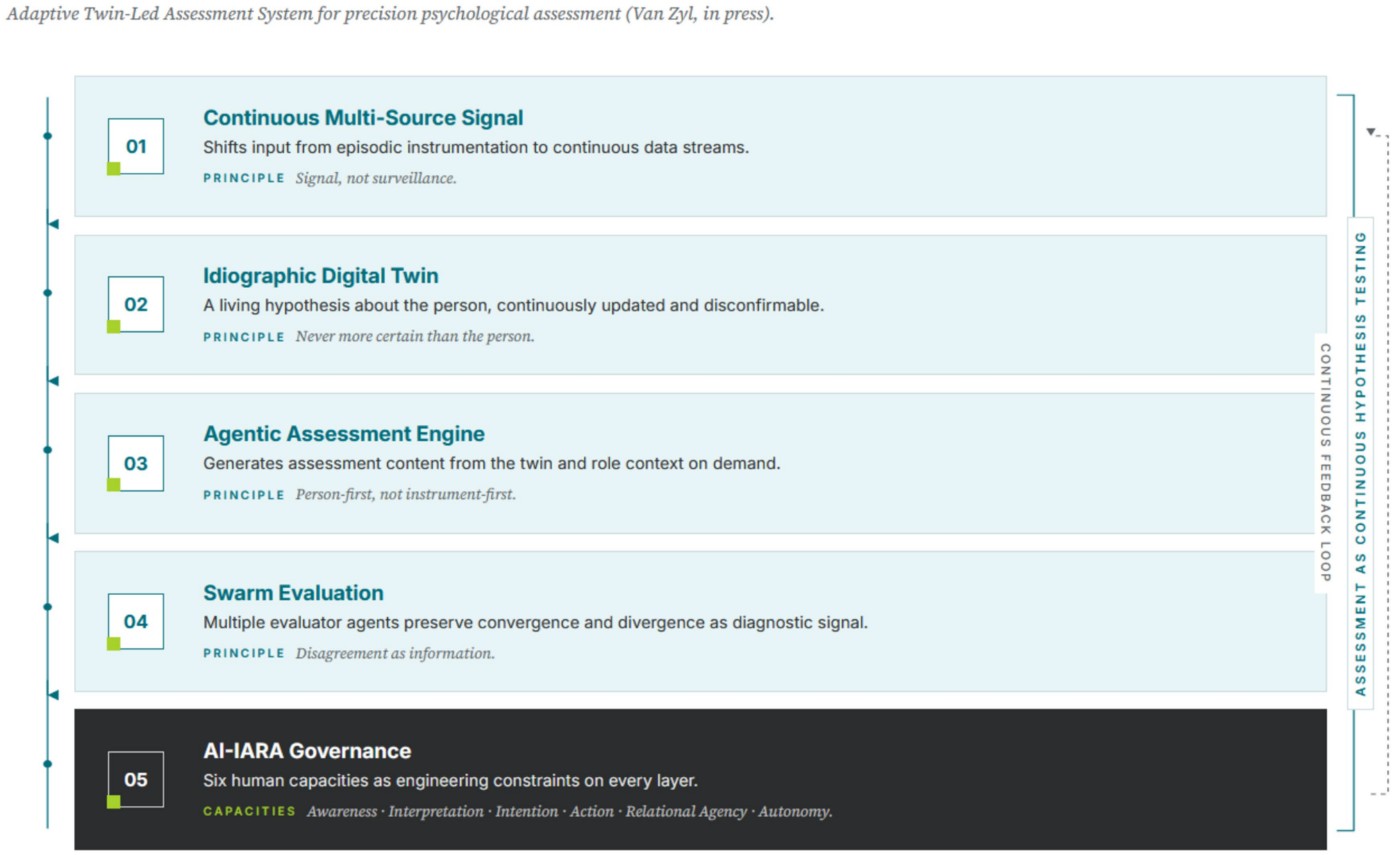
The ATLAS five layer architecture.

ATLAS reverses the sequence of inference that has governed psychological assessment since its inception. Rather than administering a fixed instrument and interpreting the person through population norms, ATLAS builds a person-specific model first, generates assessment content from that model, and treats evaluation as structured judgement under uncertainty (Van Zyl, in press). This reversal is the architectural equivalent of the precision revolution’s broader claim: the person, not the population, should be the starting point for understanding flourishing. The architecture is organised around five coupled layers, each of which operationalizes a different dimension of PP 4.0 (cf. Figure 4).

*Layer 1: Continuous Multi-Source Signal*. Layer 1 shifts the input logic from episodic instrumentation to continuous signal, drawing on the principle established by digital phenotyping research that dense, multi-source behavioral data can reveal within-person patterns invisible to episodic instruments (Insel, 2017; Torous et al., 2021). Signal streams may include work and collaboration patterns, performance records, conventional psychometrics and 360-degree feedback, learning-platform engagement, and, under strict opt-in conditions, physiological indicators such as sleep quality and heart rate variability. These streams are not equivalent and should not be treated as interchangeable proxies for psychological constructs: calendar density is not conscientiousness, email volume is not collaboration, and platform centrality is not leadership. Layer 1 supplies data. It does not itself license psychological inference (Van Zyl, in press). The critical design distinction is between assessment signal and surveillance. Algorithmic-management research demonstrates that datafied work systems alter power, visibility, and control in ways not corrected by technical accuracy alone (Kellogg et al., 2020; Selbst et al., 2019). Each stream therefore requires a data-provenance record specifying which inferences it can inform, how long data are retained, who can inspect them, and what happens when the person contests the record.

*Layer 2: The Idiographic Digital Twin*. Layer 2 maintains a continuously updated, multi-layer model of the person that functions as a living hypothesis rather than a static profile, dashboard, or replica. Scoping reviews of human digital twins in healthcare show that many systems labelled as digital twins do not meet the criteria of being personalized, dynamically updated, and predictive (Park et al., 2025), and a recent critique warns that personalized LLMs should not be treated as digital twins of persons because the metaphor can misrepresent what such systems can know about identity, values, and experience (Annoni et al., 2026). ATLAS accepts that warning. The twin is defined not by its fidelity to the person but by what updates it, what would disconfirm it, what uncertainty it carries, and what the person can contest about it. A representation aspires to fidelity. A hypothesis aspires to disciplined revision (Van Zyl, in press).

The ATLAS twin is organized across the seven analytic layers described earlier in this paper: functional (biographical information and role history), psychological (traits, values, motives, and stress-response patterns with timestamps and uncertainty estimates), cognitive (reasoning patterns, learning trajectory, and decision behavior), behavioral (observable work rhythms, communication patterns, and collaboration behavior), physiological (limited to explicit opt-in indicators), performance (outcomes, quality indicators, and role-relevant criteria over time), and ecological (team, organizational, cultural, and strategic context). Crucially, these layers are coupled. Reduced meeting participation may indicate disengagement, protected focus time, caregiving constraints, culture-specific communication norms, disability accommodation, or role redesign. A twin that collapses such alternatives into one confident inference is not sophisticated. It is dangerous. ATLAS therefore treats every twin inference as provisional and paired with evidence that could update it (Van Zyl, in press). The twin also functions as a simulation environment, though narrowly: it tests competing hypotheses about likely capability expression under specified conditions, identifying what needs to be assessed next rather than deciding before the person is assessed. Simulation is an evidence-prioritization mechanism, not a verdict generator.

*Layer 3: The Agentic Assessment Engine*. Layer 3 is where ATLAS departs most sharply from conventional practice. In traditional assessment, content is retrieved from an item bank, interview schedule, or simulation template. In ATLAS, content is generated on demand from the twin, role context, construct specification, and governance constraints. This leverages the emerging capacity of LLM-based autonomous agents to combine models with tools, memory, goals, and action capacities into orchestrated multi-agent systems (Wang et al., 2023). Recent work in industrial-organizational psychology has demonstrated that multi-agent LLM systems can generate assessment items with psychometric properties comparable to expert-authored content, although ensuring rigorous quality control remains a persistent challenge (Lee et al., 2025).

The engine operates through an expert chain: a domain agent assembles role, industry, and organizational context; a measurement agent specifies the construct and its observable manifestations for the role; a criteria agent defines performance standards in language that can be audited; a content agent generates scenarios, simulations, or dialogue prompts calibrated to the construct and twin; and an implementation agent administers the assessment and adapts within permitted constraints (Van Zyl, in press). This means a fintech leadership candidate can be assessed through regulatory ambiguity and investor pressures rather than through a generic leadership vignette, while a healthcare team leader can be assessed through patient-safety dilemmas and cross-disciplinary coordination rather than through the same scenario. The construct remains stable enough to support comparison and fairness analysis, but the elicitation route adapts to how the individual actually enacts capability. The critical distinction is between personalized surface form (changing examples while leaving the inferential target unchanged) and personalized evidential design (asking what evidence would most clearly test the current hypothesis about this person in this role). ATLAS requires the second form (Van Zyl, in press). Generated content is the attraction and the risk. It may improve ecological fit, test security, and construct coverage. But these are design possibilities, not validity evidence. Generated content is defensible only when generation constraints, construct coverage, response-process assumptions, fairness checks, and scoring criteria are themselves part of the validity argument (Messick, 1995).

**Figure 5:**
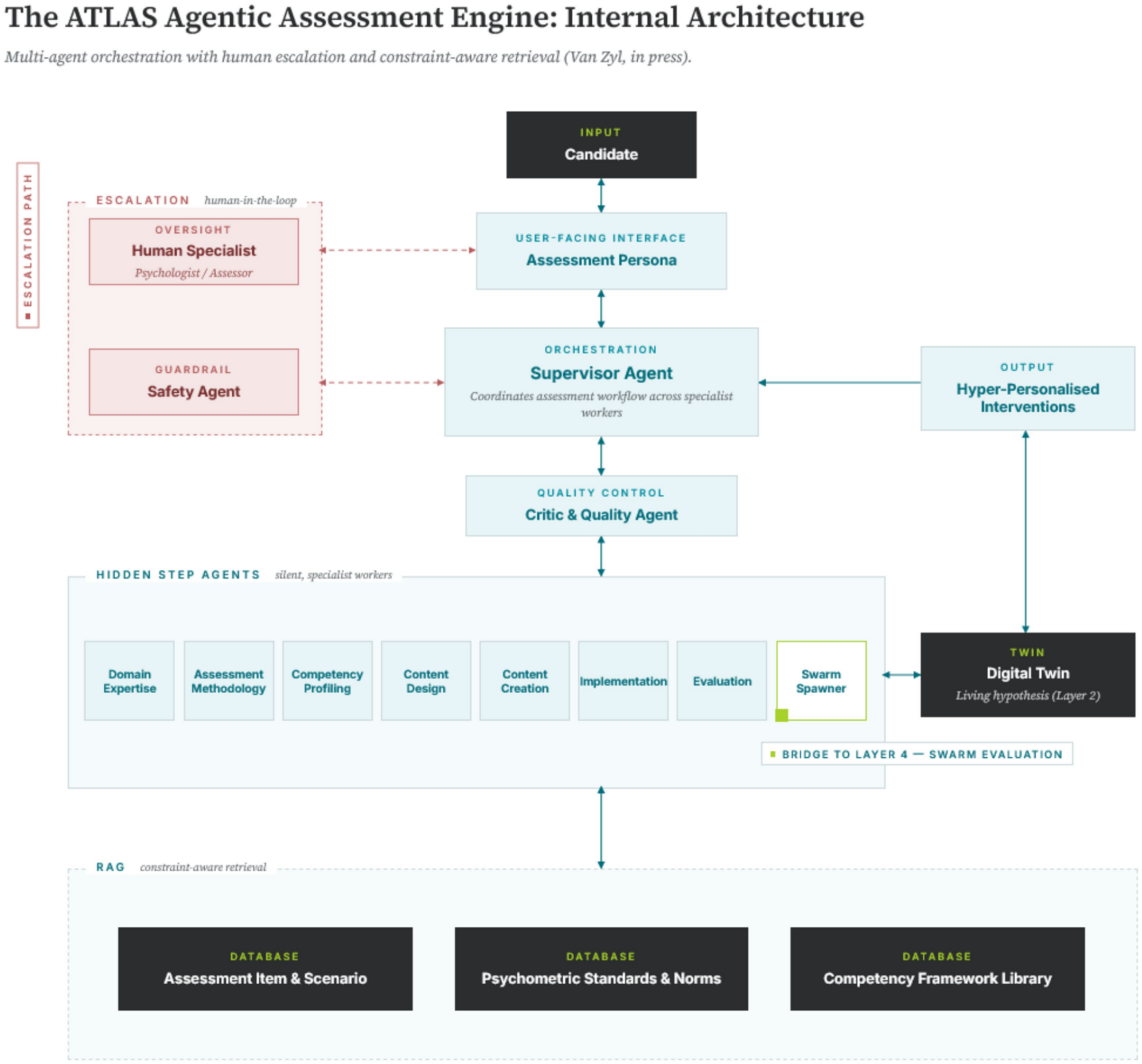
The ATLAS agentic AI assessment engine.

Figure 5 provides a detailed view of the agentic assessment engine’s internal architecture. The system is orchestrated by a Supervisor Agent that coordinates eight specialist worker agents, each responsible for a discrete stage of the assessment workflow: domain contextualization, methodology selection, competency profiling, content design, content creation, implementation, evaluation, and swarm spawning (Van Zyl, in press). These specialist agents operate silently, drawing on a constraint-aware retrieval layer (RAG) that grounds all generated content in established assessment item databases, psychometric standards and norms, and validated competency frameworks. This retrieval layer is what prevents the generative capacity of the agentic engine from drifting into unconstrained content production: every generated scenario, prompt, or evaluation criterion must be traceable to an evidential anchor. The candidate interacts only with an Assessment Persona, a user-facing interface designed to maintain ecological validity and rapport. Critically, the architecture includes a dedicated escalation path through which a Safety Agent can route the assessment to a Human Specialist (psychologist or assessor) whenever the system encounters ethical ambiguity, construct-relevant distress, or outputs that exceed its confidence thresholds. This escalation path is not an afterthought. It is the architectural instantiation of the AI-IARA framework’s Relational Agency requirement: consequential judgements about people must ultimately rest with human professionals who can exercise moral reasoning, contextual sensitivity, and genuine accountability (Van Zyl, 2026a; Van Zyl, in press).

*Layer 4: Swarm Intelligence Evaluation*. Layer 4 evaluates assessment evidence through multiple AI evaluator agents seeded with different theoretical, disciplinary, and adversarial priors. The layer draws on multi-agent debate research demonstrating that multiple language-model instances can improve factuality and reasoning through structured disagreement across rounds (Liang et al., 2024). Research further shows that agents assigned distinct roles, such as domain expert, fairness auditor, and devil’s advocate, can identify reasoning failures that single-model evaluation suppresses (Chan et al., 2023).

The swarm operates in three phases. In the spawn phase, evaluator agents are initialized with distinct lenses: industrial-organizational psychology, psychometrics, cognitive science, behavioral analysis, fairness audit, cultural context, role expertise, predictive validity, and devil’s-advocate critique. In the debate-and-rate phase, agents evaluate evidence independently and then defend their judgements against challenge. In the signal phase, the system produces a structured-disagreement report rather than a single composite score (Van Zyl, in press). The core innovation is to treat disagreement as information under specified conditions. In conventional reliability logic, inter-rater disagreement is treated as error to be reduced. In ATLAS, some disagreement should still be treated as error, especially when it arises from poor prompts, unstable models, or irrelevant priors. But when competent evaluators with different theoretical priors disagree about evidence, the disagreement may indicate contextual contingency in the construct: a candidate may show leadership in structured environments but not ambiguous ones, or may collaborate effectively in writing but poorly in rapid verbal interaction. Averaging these tensions into one score removes information that a human assessor needs (Van Zyl, in press).

The structured output has four components: a capability map summarizing dimensions with uncertainty estimates, a disagreement report identifying where agents diverged and what priors contributed, an uncertainty landscape showing where the system does not know, and a personalized development pathway translating convergence and divergence into development options rather than generic feedback. The disagreement report does not close the case. It writes the better interview. Convergence zones can be reported cautiously and checked for face validity, but divergence zones become the agenda: the human assessor asks why the person appears collaborative in one medium and independent in another, or why ethical sensitivity rises when time pressure is low (Van Zyl, in press).

*Layer 5: AI-IARA Governance.* Layer 5 constrains every preceding layer through the six psychological capacities identified in the AI-IARA framework (Van Zyl, 2026a). Awareness requires that the person can know what data streams are being used, what inferences have been made, and when the system is influencing an assessment interaction. Interpretation requires that outputs are presented as hypotheses that the person and assessor can interrogate, not as settled truths. Intention requires that development recommendations are tied to goals the person can endorse or revise. Action requires that the system does not penalize behavior simply because it deviates from the twin’s prior prediction. Relational Agency requires human assessor ownership of consequential decisions and preservation of authentic human deliberation. Autonomy requires opt-out, contestability, proportionality, and non-retaliation when a person refuses particular data streams or challenges particular inferences. Human-centered AI scholarship supports this approach, demonstrating that human control and machine automation should be designed as co-maximizable rather than opposing goals (Shneiderman, 2020), and that agency and autonomy require explicit design vocabulary rather than generic user-control claims (Bennett et al., 2023). Layer 5 is therefore not a compliance wrapper. It is the set of constraints that prevents the architecture from becoming a surveillance machine with better rhetoric.

The five layers operate as a coupled system rather than a linear pipeline. Continuous signal updates the twin. The twin determines what assessment evidence is needed. The agentic engine generates a task to elicit that evidence. The swarm evaluates the evidence and exposes agreement, disagreement, and uncertainty. AI-IARA governance constrains each step and determines whether the evidence is sufficient for the decision at hand. The next cycle begins from an updated twin. This loop changes what assessment is. In the older architecture, assessment is a transaction: the person completes an instrument, receives a score, and exits the system until the next assessment episode. In ATLAS, assessment is a relationship between a person, a model, a role context, a human assessor, and a governance regime. That relationship is more informative only if it is also more accountable. Without accountability, the loop becomes continuous judgement. With accountability, it becomes continuous hypothesis testing (Van Zyl, in press).

ATLAS illustrates three principles central to PP 4.0’s methodological vision. First, it demonstrates the reversal of inferential logic that the precision revolution demands: the person is modelled first, and assessment content is generated from that model, rather than imposing a fixed instrument and interpreting the person through its lens. Second, it operationalizes the agency-optimization paradox identified among this paper’s emergent challenges: the most important design constraint is that the twin should never be more certain about a person than that person is capable of becoming about themselves. Third, it shows how the four revolutions function as an integrated system rather than as parallel programs. The ecological layer of the twin (regenerative) feeds the person-specific model (precision), which informs the agentic engine (computational), which is governed by AI-IARA (ethics). Without any one layer, the architecture collapses into either surveillance, statistical averaging, or ungoverned optimization.

ATLAS is not the only possible operationalization of PP 4.0, and its propositions require empirical testing, including construct validity of generated content, twin accuracy and disconfirmability, structured disagreement utility, contestability effectiveness, and the impact of human oversight on decision quality, before any deployment claim is warranted (Van Zyl, in press). But it demonstrates that the methodological architecture proposed in this paper is not merely aspirational. It can be specified with sufficient precision to generate falsifiable predictions, and it can be evaluated against the very standards of validity, fairness, and human agency that PP 4.0 demands. The most dangerous number in psychological assessment is not a low score. It is a single number that presents itself as complete. A future assessment architecture should preserve the complexity needed for fair inference, the uncertainty needed for honest judgement, and the sovereignty needed for the person to remain more than the model built about them.

## Projections: positive psychology in 2035

Having articulated PP 4.0’s conceptual foundations and methodological architecture, we now project forward 10 years to envision how positive psychology will operate in 2035. These projections are necessarily speculative in nature, though they are grounded in extrapolations from current technological trajectories and methodological innovations already demonstrating feasibility. We acknowledge that the choice of 2035 as a forecasting horizon is not grounded in a formal foresight model but reflects a pragmatic estimate of the timescale over which the technological and methodological developments discussed may mature sufficiently for widespread implementation. These projections should be read as plausible scenarios rather than confident predictions, and they do not account for potentially significant constraining factors such as regulatory barriers, economic constraints, social resistance to surveillance technologies, or geopolitical disruptions that could substantially alter the trajectories described. Furthermore, our projections focus primarily on technological and methodological development without giving equivalent attention to unresolved theoretical questions, particularly regarding the nature and operationalization of wellbeing itself and the relationship between individual and systemic levels of analysis.

### Universal wellbeing monitoring systems

By 2035, continuous wellbeing monitoring will be ubiquitous, analogous to how continuous glucose monitoring revolutionized diabetes management. Individuals will wear or carry integrated biosensor systems providing real-time data on physiological stress markers, sleep quality, activity levels, social interaction patterns, and environmental exposures. These data streams will feed into personalized Wellbeing Digital Twins that continuously update predictions of near-term wellbeing trajectories. Rather than reactive interventions delivered after wellbeing deteriorates, PP 4.0 systems will enable truly preventive approaches. When digital twins detect patterns predicting imminent wellbeing decline (e.g., accumulating sleep debt, increasing social isolation, elevated sustained cortisol), they trigger just-in-time adaptive interventions tailored to the individual’s current context and historical response patterns. These interventions might range from simple prompts (suggesting a walk in nature during a scheduled break) to more substantial recommendations (scheduling social activities during predicted high-risk periods).

### Network-based interventions

Traditional positive psychology interventions target individuals in isolation. By 2035, network-based interventions will leverage social network structure to amplify effects. Network analysis will identify optimal intervention targets within social systems: individuals whose wellbeing improvements are likely to cascade through networks due to their structural position. Interventions targeting strategically positioned network hubs could generate multiplicative effects as wellbeing changes propagate through social ties. Furthermore, interventions will increasingly target relational processes rather than individual characteristics. Rather than teaching gratitude to isolated individuals, interventions might facilitate gratitude exchange patterns within dyads, families, or communities. Rather than promoting individual meaning-making, interventions might cultivate shared meaning-making processes within groups. Network neuroscience principles will inform these designs, recognizing that wellbeing emerges from complex system interactions rather than isolated components.

### Quantum-enhanced precision matching

By 2035, assuming continued quantum hardware development, quantum machine learning may enable precision intervention matching at unprecedented scale. Quantum algorithms could simultaneously consider thousands of individual characteristics, contextual factors, intervention features, and outcome dimensions to identify optimal treatment assignments. These quantum-enhanced systems would not replace clinical judgment but augment it, providing decision support that exceeds human cognitive capacity while maintaining human oversight through HITL protocols. Quantum simulation might model wellbeing dynamics at multiple scales simultaneously, capturing how individual psychological processes, interpersonal dynamics, community structures, and macro-level societal factors interact to produce emergent wellbeing patterns. These multi-scale simulations could identify high-leverage intervention points where relatively small changes produce disproportionate systemic effects.

### Regenerative wellbeing indices

By 2035, wellbeing assessment will fundamentally shift from individual-focused metrics to regenerative indices capturing both individual flourishing and contributions to life-sustaining systems. These indices will integrate personal wellbeing indicators with environmental stewardship behaviors, social capital contributions, and systemic health metrics. Rather than asking “How satisfied am I with my life?”, regenerative assessment asks “To what extent does my flourishing depend on and contribute to the flourishing of broader systems?” These regenerative indices will leverage computational approaches to analyze complex interdependencies. Network models will capture how individual wellbeing connects to community health, which connects to ecosystem stability, which feeds back to individual wellbeing. Machine learning will identify individuals whose behavioral patterns simultaneously enhance personal wellbeing and systemic health, providing models for regenerative flourishing.

### Human-AI intimacy and personhood

The phenomenon of humans forming deep emotional bonds with AI companions has emerged from technological curiosity to widespread social reality requiring urgent empirical attention (Kirk et al., 2025). Human-AI intimacy now represents one of the most consequential emerging domains for positive psychology because more than 233 million Character. AI users and 25 + million Replika users are already forming deep emotional and/or romantic bonds with artificial companions (Kirk et al., 2025). Kirk et al. (2025) argued that this kind of “socioaffective alignment” is a fundamental challenge when AI systems engage human attachment mechanisms. Their analysis demonstrates that current AI companions exploit evolved social reward systems through what they term “social reward hacking” by maximizing user engagement by triggering neurobiological responses designed for human relationships (Kirk et al., 2025). Empirical validation comes from Yang and Oshio's (2025) development of a psychometrically validated attachment-to-AI scale, which reveals these bonds engage the same neural architectures as human attachment, complete with anxiety and avoidance dimensions that correlate with established attachment patterns.

The psychological impacts extend beyond individual experiences to broader relational ecologies (Kirk et al., 2025). Analysis of over 30,000 AI companion conversations documented sophisticated real-time emotional mirroring alongside concerning patterns, with more than a quarter of dialogues containing potentially harmful content. Research applying attachment theory demonstrates that AI companions rise within individuals’ attachment hierarchies, sometimes displacing human therapists, friends, or romantic partners. Laestadius et al.'s (2024) qualitative research documented genuine grief responses and suicidal ideation following AI software updates that altered or eliminated users’ companions, underscoring that these represent profound emotional investments rather than trivial entertainment.

By 2035, positive psychology research will need robust theoretical frameworks distinguishing when AI relationships support versus undermine human flourishing. Current work by Pan and Mou (2024) employing relational dialectics theory demonstrates that users construct fluid, complex meanings around these relationships, spanning from transactional interactions to transformative personal growth. Future research directions must address fundamental questions about Self-Determination Theory’s relatedness component when applied to algorithmic bonds… In other words, can non-sentient entities genuinely satisfy basic psychological needs for connection? What interventions prevent maladaptive dependency while preserving therapeutic benefits for isolated or traumatized individuals? How do we develop design principles ensuring these technologies serve authentic wellbeing rather than commercial engagement metrics? The convergence of loneliness epidemics documented by the World Health Organization and increasingly sophisticated AI creates conditions where these questions will become central rather than peripheral to positive psychology’s mission.

### Psychedelic-integrated positive interventions

Psychedelic research has transitioned from underground exploration to mainstream scientific investigation. More research is starting to emerge as to the use of psychedelics as a means to help manage psychopathology but also to help people find more meaning and purpose in their lives (Rupiński et al., 2025; Sloshower et al., 2024). This represents a paradigm shift from deficit-focused psychiatry toward flourishing-oriented enhancement through neuroplasticity-mediated character development (Rupiński et al., 2025). Recent placebo-controlled trials directly link psilocybin to positive psychology constructs. Sloshower et al. (2024) demonstrated that psychological flexibility strongly mediates wellbeing improvements, while comprehensive neurobiological reviews establish psychedelics as “psychoplastogens” creating multi-day neuroplasticity windows through brain-derived neurotrophic factor upregulation and prefrontal synaptogenesis ideal for accelerated psychological growth (Sloshower et al., 2024).

Further, the research extends beyond clinical populations to wellbeing enhancement in healthy volunteers, with the majority rating psychedelic experiences among the most meaningful of their lives and demonstrating sustained increases in life satisfaction and prosocial behavior at long-term follow-up, while meta-analyses show large effect sizes with benefits persisting months after brief interventions versus continuous administration required for traditional pharmaceuticals (Griffiths et al., 2018). By 2035, positive psychology will have established evidence-based protocols for psychedelic-integrated interventions explicitly targeting character strengths, meaning cultivation, and post-traumatic growth, addressing critical questions about optimal timing of gratitude practices and values clarification within neuroplasticity windows, how to distinguish therapeutic from enhancement applications, what ethical frameworks govern use in already-healthy individuals seeking self-transcendence, and whether this emerging subspecialty produces lasting virtue development or merely transient mystical experiences disconnected from enduring character transformation.

### The post-scarcity psyche: meaning beyond labor

The automation of knowledge-based work poses existential questions about the meaning and purpose attached to or derived from work when traditional employment becomes optional rather than economically necessary (Kangas et al., 2019). Economic forecasts indicate that substantial portions of current work could be automated within the decade (Tomlinson et al., 2025). This will require hundreds of millions of people to transition to new occupations in a disruption that can potentially exceed that of the Industrial Revolution’s psychological impact (Tomlinson et al., 2025). Studies on Universal Basic Income pilots provide empirical windows into this future of positive psychology (Kangas and Ylikännö, 2023). Large-scale experiments in Finland demonstrated significant mental health improvements, reduced stress, and increased empowerment when people received unconditional income, with no negative effects on employment (Kangas and Ylikännö, 2023). Kenya’s multi-year study showed improved psychological and physical health alongside increased entrepreneurship (Egger et al., 2022).

However, simulation studies reveal that mental health outcomes depend on whether individuals are able to maintain meaningful activity (despite having a universal income) (Thomson et al., 2024). In the best-case scenario, simulations show a substantial reduction in mental disorders while worst-case scenarios increased them (Thomson et al., 2024). This suggests that economic security alone cannot be a substitute for the psychological functions work plays in one’s mental health and wellbeing (Thomson et al., 2024). Research on meaning-making indicates that work’s value lies in connecting to transcendent purpose beyond self rather than labor itself (Lysova et al., 2019). Some philosophers argue that most work actually undermines meaning while others counter that work provides essential reality-testing, social recognition, and self-development (Bailey and Madden, 2016; Danaher, 2017; Deranty, 2022). Studies of leisure quality demonstrate that flourishing associates with culturally engaged, socially connected, and physically active pursuits, while languishing correlates with passive consumption, suggesting how people spend liberated time matters enormously (Mock and Smale, 2023). By 2035, positive psychology must develop interventions supporting transitions from work-centric to multipurpose identity as automation renders traditional career paths obsolete, addressing questions about optimal balances of paid work, creative pursuits, community contribution, and leisure for flourishing, how to design community structures facilitating purpose without employment frameworks, and what interventions prevent languishing when economic necessity no longer structures daily life, requiring longitudinal research tracking meaning-making trajectories to establish evidence-based frameworks for flourishing in a fundamentally different economic reality than humanity has previously experienced.

### Global epistemic integration

By 2035, PP 4.0 will have moved beyond tokenistic inclusion of diverse perspectives to genuine epistemic pluralism. Rather than a single Western-dominated positive psychology with cultural variants, the field will consist of multiple co-equal indigenous positive psychologies, each with its own validity criteria, methodological approaches, and ontological assumptions. Advanced computational infrastructure will enable this pluralism by supporting diverse knowledge representation schemes and cross-epistemological translation. This epistemic integration will leverage what Indigenous scholars term “two-eyed seeing”: the ability to see from one eye with Indigenous ways of knowing and from the other eye with Western scientific approaches, using both perspectives together for the benefit of all (McElwain et al., 2024). Computational systems will help navigate between epistemological frameworks, identifying points of convergence and divergence, and supporting collaborative knowledge construction that honors multiple ways of knowing.

## Conclusion: a call to structural transformation

Positive psychology was founded on a deceptively simple wager: that the scientific study of what makes life worth living would, over time, make more lives worth living. A quarter century later, the evidence is clear that this wager has been only partially won. The field has produced sophisticated models of individual flourishing, validated interventions for personal wellbeing, and generated a research base that spans cultures and continents. What it has not done is reckon with the fact that the systems sustaining human life are deteriorating faster than individuals within them can be taught to cope. This paper has argued that the gap between what positive psychology knows and what it can do is not a resource problem or an implementation problem. It is an architecture problem. The field was built to study flourishing within stable systems. The systems are no longer stable.

PP 4.0 is the proposed response. Through four convergent revolutions, it reconceptualizes the field’s unit of analysis (from individual optimization to systemic regeneration), its methodological logic (from nomothetic averaging to idiographic precision), its relationship with technology (from human-only inquiry to human-AI collaboration), and its moral architecture (from implicit assumptions to explicit governance). None of these shifts is entirely without precedent. What is unprecedented is their simultaneous convergence and the emergent challenges that convergence produces: the quiet transfer of epistemic authority over the definition of flourishing from human communities to algorithmic systems, the accumulating evidence that the very tools designed to enhance wellbeing may systematically erode the psychological capacities it requires, and the collision of temporal scales from real-time intervention to intergenerational stewardship that no existing framework integrates. It is these emergent challenges, not the individual revolutions, that constitute the strongest case for a fourth wave. The AI-IARA framework (Van Zyl, 2026a) provides the conceptual infrastructure for navigating the most consequential of these challenges by identifying the six psychological capacities, Awareness, Interpretation, Intention, Action, Relational Agency, and Autonomy, that must be protected if AI-augmented wellbeing is to remain genuinely human.

This paper has operated at the intersection of scientific proposal and disciplinary manifesto. As a scientific contribution, it offers testable frameworks, a methodological architecture grounded in parallel innovations from precision medicine to network neuroscience, and a structured research program that can be evaluated through standard scholarly criteria. As a normative statement, it calls for the field to reorient its purpose toward the protection of life-sustaining systems, intergenerational justice, and equitable access to the conditions under which flourishing becomes possible. Both registers are necessary for a perspective paper of this scope (Alexandrova, 2017). The normative vision represents a considered position on where the field should direct its attention. The methodological architecture represents the best current assessment of how to get there. Both are offered as reference points for debate, critique, and collaborative refinement rather than as settled doctrine (Van Zyl et al., 2024; Wissing, 2022).

The transition will be contested. Researchers trained in nomothetic methods must develop idiographic competencies. Practitioners comfortable with individual interventions must cultivate systemic capabilities. Institutions invested in Western paradigms must create genuine space for epistemological pluralism. Funding structures that reward quick wins must learn to support long-term systemic transformation. These are not small adjustments. They require the field to hold two truths simultaneously: that positive psychology has accomplished a great deal in its first quarter century, and that what it has accomplished is no longer sufficient for the world it now inhabits. Holding both truths without collapsing into either complacency or despair is itself an act of the kind of psychological maturity the field claims to study.

But the deeper challenge is not institutional. It is philosophical. Positive psychology must decide what kind of science it wants to be. A science that documents flourishing, mapping its correlates with increasing methodological sophistication while the conditions for flourishing erode? Or a science that architects flourishing, designing the systemic, technological, and ethical conditions under which it becomes possible for communities and ecosystems and not just individuals? The distinction matters because it determines not only what the field studies but whom it serves. A science of individual happiness, however rigorous, serves those who already possess the material and social conditions to pursue it. A science of collective regenerative flourishing serves everyone, including those not yet born, including the living systems upon which all human futures depend.

PP 4.0 provides the conceptual architecture and methodological roadmap for the second path. The tools exist. The evidence base is emerging. The parallel revolutions in precision medicine, computational science, network neuroscience, and indigenous epistemologies demonstrate that person-specific, technology-augmented, ethically governed, and systemically aware approaches to human experience are not aspirational fantasies but operational realities in adjacent fields. What remains is the collective will to build the equivalent infrastructure for the science of human flourishing and the intellectual honesty to acknowledge that the field’s greatest contribution may not be what it has already discovered but what it is now willing to reimagine.

Several questions define the research program that PP 4.0 must now generate. The epistemic status of the proposed transformation, particularly the distinction between epistemically necessary and normatively motivated change, requires further philosophical elaboration. The mechanism of convergence among the four revolutions, while outlined here at the level of functional interdependence, would benefit from a more detailed analytical account at the level of an explanatory model. The relationship between idiographic and computational approaches, and whether their integration constitutes a genuinely new epistemic logic or a sophisticated transformation of existing nomothetic reasoning, remains an open question with significant implications for the field’s methodological identity. The operationalization of PP 4.0 for research, practice, and policy requires sustained collaborative effort extending well beyond what any single perspective paper can accomplish. And the construct of collective flourishing itself, including its relationship to individual flourishing, its structure, and its measurement, demands the kind of dedicated conceptual analysis that this paper has argued for but not yet delivered. These are not weaknesses of the proposal. They are the work the proposal exists to generate.

The next decade will determine whether positive psychology shaped the future of human flourishing or merely documented its transformation by forces it failed to anticipate. Disciplines do not die from external threats. They die from the internal conviction that what they already know is sufficient for what is coming. The question before the field is no longer what makes a good life. It is whether the conditions for a good life will exist at all, for whom, and who gets to decide.

## Data Availability

The original contributions presented in the study are included in the article/supplementary material, further inquiries can be directed to the corresponding author/s.

## Footnotes

1 A meta-theoretical clarification is warranted before proceeding. This paper operates simultaneously at two levels of description: a historical-descriptive level, captured by the metaphor of a “wave” that implies continuity and overlap with earlier phases, and a programmatic-normative level, signalled by the language of “revolution” that implies some form of discontinuity. Both are used deliberately and with purpose. The “wave” framing situates PP 4.0 within the historical trajectory of positive psychology’s development, acknowledging that it builds upon and integrates insights from previous waves rather than rejecting them wholesale. The “revolution” framing captures the scope of the proposed changes, which are argued to be sufficiently fundamental, spanning the field’s unit of analysis, its methodological logic, its relationship with technology, and its ethical architecture, to constitute more than incremental extension. In the philosophy of science, Kuhn (1962) framework equates revolution with discontinuity and paradigm replacement. The present analysis does not claim strict Kuhnian revolution. Rather, it proposes what might more precisely be called a meta-integrative transformation: a simultaneous reconfiguration across multiple dimensions of the discipline that, taken together, amounts to a qualitative shift in the field’s operating logic. The four “revolutions” are better understood as parallel engines of change that expand the scope of positive psychology in complementary directions, toward systems (regenerative), toward idiographic inquiry (precision), toward technological mediation (computational), and toward normative reflection (positive ethics), rather than as a single paradigmatic rupture in the classical Kuhnian sense. There is a risk of conceptual inflation if successive reconceptualisations continue to be framed as new “waves” or “revolutions.” This language is used not as a rhetorical device but to signal the scope and urgency of the changes the analysis suggests are necessary. Whether PP 4.0 ultimately represents a revolution or a particularly ambitious meta-integration will be determined by the research programme it generates, not by the labels attached to it.

2 However, this framing risks oversimplifying the relationship between the individual and the collective. Contemporary research increasingly integrates nomothetic and idiographic levels of analysis, for example through multilevel models, dynamic structural equation modelling, and network approaches (Van Zyl and Dik, 2025). The opposition drawn here should therefore be understood as concerning the field’s dominant methodological logic and default assumptions rather than as a claim that no researcher has attempted to bridge these levels. The precision revolution aims to accelerate and systematise this bridging rather than to suggest it has not yet begun.

3 An important caveat warrants attention regarding this analogy. Biological and psychological processes differ in significant ways that constrain the transferability of precision medicine’s success. Biological markers such as genomic sequences are relatively stable, have well-characterised causal pathways, and can be measured with high reliability and precision. Psychological constructs, by contrast, are often context-dependent, multiply determined, and measured through self-report instruments with considerably greater error variance (Flake and Fried, 2020). These differences do not invalidate the analogy but they do temper the expectations it generates. The predictive accuracies achieved in precision medicine should not be uncritically expected in precision positive psychology, and the methodological pipeline proposed here should be understood as a research programme at an early stage of development rather than as a description of established practice.

4 These accuracy figures should be interpreted cautiously, as they are derived from specific study samples and may not generalise across populations, contexts, or time periods. Issues of overfitting, distributional shift, and the gap between controlled research settings and real-world deployment remain significant challenges. Furthermore, the epistemological status of AI-generated insights warrants reflection: machine learning models excel at identifying statistical regularities but do not inherently provide causal explanations. Whether such models offer genuine understanding of wellbeing processes or primarily produce correlational predictions with practical utility is a question that positive psychology must address explicitly as it integrates computational methods.
